# Diversification of the Alpine Chipmunk, *Tamias alpinus,* an alpine endemic of the Sierra Nevada, California

**DOI:** 10.1186/1471-2148-14-34

**Published:** 2014-02-23

**Authors:** Emily M Rubidge, James L Patton, Craig Moritz

**Affiliations:** 1 Museum of Vertebrate Zoology, 3101 Valley Life Sciences Building University of California, Berkeley, California 94720-3160, USA; 2 Royal British Columbia Museum, 675 Belleville Street, Victoria, British Columbia V8W 9W2, Canada; 3 Research School of Biology, The Australian National University, Acton, ACT 0200, Australia

## Abstract

**Background:**

The glaciation cycles that occurred throughout the Pleistocene in western North America caused frequent shifts in species’ ranges with important implications for models of species divergence. For example, long periods of allopatry during species’ range contractions allowed for the accumulation of differences between separated populations promoting lineage divergence. In contrast, range expansions during interglacial periods may have had homogenizing effects via increased gene flow following secondary contact. These range dynamics are particularly pronounced in the Sierra Nevada, California, given the complex topography and climatic history of the area, thus providing a natural laboratory to examine evolutionary processes that have led to the diversity patterns observed today.

**Results:**

Here we examined the role of late Pleistocene climate fluctuations on the divergence of the Sierra Nevada endemic Alpine Chipmunk (*Tamias alpinus*) from its sister taxon, western populations of the Least Chipmunk (*T. minimus*) from the Great Basin. We used one mitochondrial gene (*cytochrome b*) and 14 microsatellite loci to examine the evolutionary relationship between these species. Mitochondrial sequence data revealed that *T. alpinus* and *T. minimus* populations share mitochondrial haplotypes with no overall geneaological separation, and that diversity at this locus is better explained by geography than by species’ boundaries. In contrast, the microsatellite analysis showed that populations of the same species are more similar to each other than they are to members of the other species. Similarly, a morphological analysis of voucher specimens confirmed known differences in morphological characters between species providing no evidence of recent hybridization. Coalescent analysis of the divergence history indicated a late Pleistocene splitting time (~450 ka) and subsequent, though limited, gene flow between the two lineages.

**Conclusions:**

Our results suggest that the two species are distinct and there is no contemporary introgression along their geographic boundary. The divergence of *T. alpinus* during this time period provides additional evidence that Pleistocene glacial cycles played an important role in diversification of species in Sierra Nevada and North America in general.

## Background

Understanding processes that promote and maintain biodiversity is a key goal of evolutionary biology. Divergent natural selection resulting from resource heterogeneity and competitive interactions can drive population divergence and speciation [1,2]. Nonadaptive divergence, operating via genetic drift due to isolation and founder effects, may also play a significant role in generating patterns of species diversity. Furthermore, hybridization (or reticulate evolution) during and subsequent to speciation can add novel genetic diversity to diverging lineages and affect the course of adaptive divergence [3-5]. The cyclical Pleistocene glacial and interglacial episodes have shaped the genetic architecture of taxa across the globe, as retraction to refugia facilitated the formation of distinct evolutionary lineages within species [6,7] through both vicariant processes and shifting the spatial distribution and extent of ecological gradients. Recurrent fragmentation and expansion provides opportunities for initial divergence, repeated secondary contact, hybridization, and demographic fluctuation [8].

Range dynamics and the complex geological and climatic history of the Sierra Nevada, California has shaped diversity patterns of the central-western United States over the time span of most, if not all, extant species. The late Pleistocene in particular was a time of drastic climatic fluctuations in the Sierra Nevada [9] and the adjacent Great Basin and Mohave biomes [10] and thus a period linked to both intra- and interspecific diversification in many taxa (e.g., [11-14]).

### The physical setting: Sierra Nevada and the Great Basin

The Sierra Nevada and the adjacent Great Basin is a biologically diverse region with a rich glacial history [10,15-17]. The Sierra Nevada are a narrow, elongated, topographically complex, high, and relatively young mountain range that spans about 640 km from north to south and 110 km from west to east, and contains the highest peak in the continental United States (Mt. Whitney, 4,421 m). Given the steep elevational gradient on the east side of the Sierra Nevada, the mountain range casts a major rain shadow affecting the climate and ecology of the adjacent central Great Basin of the intermountain west. The Great Basin consists of over 10,500 km^2^ of valleys, basins, lakes and mountain ranges with extreme elevational relief throughout the region. It contains the lowest point in North America (-86 m, in Death Valley, California) as well as one of the highest (4342 m, White Mountain Peak, CA) [17]. The complexity of small mammal distributions in relation to the high environmental diversity in the Great Basin, have made this region a focus for biogeographic research (e.g., [17-20]).

The interactions between Sierra Nevada with the Great Basin and Mojave Desert biomes during the cyclical glaciations of the Pleistocene created areas of recurrent isolation and secondary contact within and between species. The Sierra Nevada has experienced at least six cyclical episodes of glacial expansion and retreat, both regional and local in extent, beginning in the early Pleistocene (1.65 ± 0.7 mybp), with the most recent advance around 3.5 kbp [21], as well as three or more neoglacial advances ending with termination of the Little Ice Age, which spanned 1350 to 1850 AD [22]. These episodes are intertwined with a similarly complex history of volcanism along the eastern flank of the range over the same time period [23]. Few glaciers remain in the Sierra Nevada today, with most having shrunk by greater than half in mass and aerial extent within the latter half of the 20^th^ century [24]. This complex glacial history and associated range dynamics and steep environmental gradients in the Sierra Nevada and the adjacent Great Basin provide a natural laboratory to examine species histories including the combined effects of range fluctuations, oscillations between isolation and introgression, and adaptive divergence across environmental gradients (e.g., [25,26]).

### Chipmunk diversity in western North America

The biogeographic history, radiation, and evolutionary relationships of western North American chipmunks are complex and include several instances of historical introgression among species [27-32]. Chipmunk diversity is centered in western North America with 23 species [28,33] and diversification has been linked to shifting ranges resulting from climatic cycles and subsequent shifts in habitat preference resulting from interspecific competition and niche partitioning over elevational gradients [34-36]. In this study, we examine the evolutionary history of the Alpine Chipmunk, *Tamias alpinus*, a narrow high-elevation endemic, relative to its widespread sister species, the Least Chipmunk, *Tamias minimus.*

The divergence history of *T. alpinus* is not well understood; however, recent work has shown that *T. alpinus* and *T. minimus* are paraphyletic [32]. *T. minimus* represents a species complex and this study focuses on the western segment of the species, geographically adjacent to the range of *T. alpinus*. Previous work by Reid et al. [32] included geographically (and taxonomically) disparate sequences from *T. minimus* individuals from California, Nevada, Utah, Wyoming and Washington and clearly showed that *T. alpinus* is nested with western segments of *T. minimus scrutator*. Here, we focus on regions of allo/parapatry in the Sierra Nevada where these two species come into close proximity to better understand the evolutionary history of *T. alpinus*.

Our objective is to examine the relationship between *T. alpinus* and *T. minimus*, with the goal of gaining a better understanding of the evolutionary history of *T. alpinus*. More specifically, we focus on two questions 1) did *T. alpinus* diverge from *T. minimus* in association with late-Pleistocene glacial dynamics and 2) is there evidence for contemporary or historical introgression as reported in other species-pairs of chipmunks [30,31]. To address these questions we examine morphological characters and genetic variation and population structure at one mitochondrial gene and 14 microsatellite loci. Contrasting patterns of nucleotide variation in the mitochondrial genome with patterns of genetic variation at microsatellite markers gives us a picture of both historical and contemporary processes respectively. And although differences in morphological characters between species are well defined (see below), testing for morphological intermediacy in individuals in adjacent versus distant populations will help distinguish between genetic similarity due to recent speciation or similarity as a result of secondary contact and hybridization.

## Methods

### Study species

**Figure 1:**
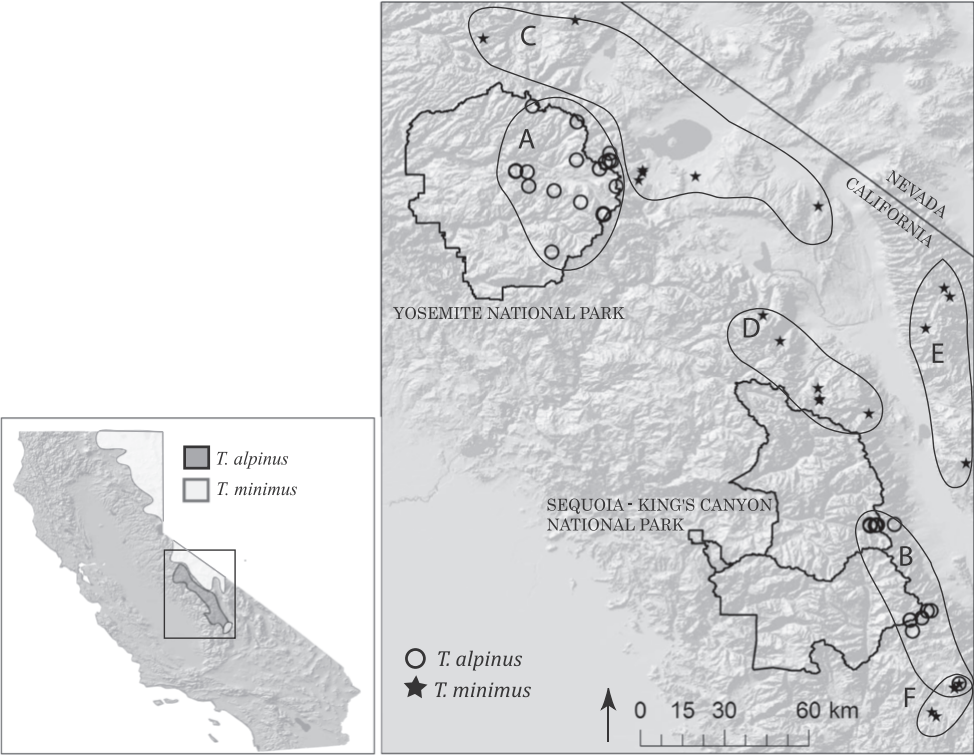
**Map of sampling localities in the Sierra Nevada, California, USA.** Open circles indicate sampling sites of *T. alpinus*, black stars show *T. minimus* sampling sites. Polygons labeled with letters show geographic groupings used in population structure analyses: A) T.alp-N; B) T.alp-S; C) T.min-N; D) T.min-C; E) T.min-Wht/Iny; F) T.min-S. Inset: Distribution of *T. alpinus* (dark gray) and *T. minimus* (light gray) in California.

The *T. minimus* species complex contains 21 recognized subspecies [37] and is the most widely distributed *Tamias* species [38]. It occurs from western central Yukon Territory southward along the eastern base of the Rocky Mountains in British Columbia (BC) eastward throughout Canada’s provinces, upper Michigan and Minnesota. It is also found throughout the Great Basin with disjunct populations further south in Arizona and New Mexico [37]. The subspecies, *T. minimus scrutator*, is the sister taxa to *T. alpinus*[32] and occurs across southeastern Oregon, south-central Washington, northern and central Nevada, western Utah, southwestern Idaho and NE California [39], mainly east of Sierras, with an isolated high elevation population at the southern terminus of the mountain range (Figure 1). For the purposes of this study, we will use *T. minimus*, to refer to the subspecies *T. m. scrutator* and specifically the Californian populations adjacent to *T. alpinus* (Figure 1).

*T. alpinus* is geographically restricted to the high elevations of the central to southern Sierra Nevada (Figure 1). Based on historical records and our own surveys, *T. alpinus* and *T. minimus* are allopatric throughout our study area. South of Yosemite, there are reported areas of sympatry in the central Sierra Nevada northwest of Bishop (D. Guiliani, pers. comm.), but none have been confirmed despite our targeted field surveys between 2009-2013. Collections made in 1911 in the southern Sierras recorded both species at the same locality (Little Brush Meadow, Tulare Co.), but apparently at different elevations (9750 ft for *T. minimus* and 10,000 ft for *T. alpinus*; field notes of the collectors, H. A. Carr and W. P. Taylor, MVZ archives). This difference in elevation signals distinctly different habitats, where lodgepole pine (*Pinus contorta*) with a sagebrush understory at the lower elevation is replaced by scattered foxtail pine (*Pinus balfouriana*) and arctic-alpine forbs near treeline. These habitats characterize the ecological distribution of the two species in other areas where their ranges come into close proximity.

The two species differ in morphology and habitat preferences. *T. minimus*, among other characters, is smaller in body mass, has a longer tail, shorter ears and darker coloration than *T. alpinus*[39] and has a different bacular (penis bone) morphology [37,40]; more specific details of these differences are summarized in Additional file 1: Table S1. In California, *T. minimus* is found in arid sagebrush habitat that ranges in elevation from 1500 m to above 3000 m in the Sierra Nevada and mountains to the immediate east (e.g., Sweetwater, White, and Inyo ranges). *T. alpinus* is restricted to the alpine zone of the Sierra Nevada at and above tree-line (2950 to 4100 m) where it occupies open granite habitat, meadow edges and talus slopes [39,41].

### Study site and samples

The study area is the central to southern Sierra Nevada, CA, USA, which includes the entire known range of *T. alpinus* and that of *T. minimus* to the immediate east and north (Figure 1). A total of 341 chipmunks were included in this study. The majority of samples were collected between 2003-2009, however 14 samples were taken from museum skins that were collected between 1911-1916 and housed in the Museum of Vertebrate Zoology (MVZ) at the University of California, Berkeley. For the samples collected between 2003-2009, we live-trapped animals using Sherman traps at 62 locations between 2003-2009 (Figure 1). We used non-lethal sampling (ear clips) and collected vouchered specimens, including liver samples, now catalogued in the MVZ (see Additional file 2: Table S4 for MVZ catalogue numbers and locations). Chipmunks collected in areas of potential sympatry were identified to species in the field based on distinct morphological differences including body size, pelage color, and ear and tail length. More rigorous morphological measurements on collected specimens were conducted in the lab to better document species-specific morphological differences. Sample collection was approved by the Animal Care and Use Committee (Protocol #R304-0509) at the University of California, Berkeley.

### Morphological methodology and analyses

Despite well-characterized differences between species, these two taxa share a close genetic legacy, and in order to distinguish between the possibility that this similarity resulted from recent common ancestry versus reticulation subsequent to divergence (e.g., [30,31,42]), we examined the morphology of specimens taken from geographically adjacent versus distant localities to test for morphological intermediacy, using the same geographic groupings in the molecular comparison (Figure 1). We examined three data sets separately. The first containing external body metrics that have been used to separate the two species including: a) color and color pattern (b) tail length and bushiness; (c) ear length (N = 36 and 129 for *T. alpinus* and *T. minimus* respectively). The second dataset was based on comparing bacular dimensions (N = 15 and 33; shaft length, mid-shaft height, tip height, tip angle, and keel breadth following [43]). The third and final dataset examined craniodental features measured from preserved skulls (N = 161 and 159). We used separate conical variate analyses (CVA; JMP 5.1.1 statistical software) for each dataset, which generates a classification matrix of group membership, with individual posterior probabilities, based on multivariate discriminant functions.

### DNA extraction, sequencing, and microsatellite genotyping

#### DNA extraction

To extract DNA from the liver or ear tissue samples, we used the standard Qiagen DNAeasy kit following the manufacturer’s protocol (Qiagen). Tissue extractions were eluted in a total of 400 μl AE buffer. All extractions and PCR set-up on the 14 skin samples from museum specimens (“historical” samples) were conducted in a separate laboratory devoted to ancient DNA research. We followed the museum skin DNA extraction protocol described in Mullen and Hoekstra [44]. After removing an approximately 3 mm × 3 mm square piece of skin from the lower lip, we placed the sample in 95% ethanol and refreshed the ethanol roughly every 3 hours over a 24-hour period to wash the sample of salts and PCR inhibitors. Following these washes, each skin sample was carefully removed of hair and shaved into smaller pieces with a scalpel and placed into a 1.5 ml locking Eppendorf tube. Between each sample, the forceps and scalpel were washed in 10% bleach, rinsed in 95% ethanol and flamed to avoid cross contamination. We extracted DNA using a Qiagen DNeasy Tissue Extraction kit with the following modifications. First we diluted the AE Buffer to 1:10 in RNAse-free H_2_O and warmed it to 70°C prior to elution. Second, we applied two elutions of 50 μl of warm 1:10 AE Buffer to the spin columns and allowed this elution step to incubate at room temperature for 5 min prior to the final spin. We conducted a negative extraction (sterilized forceps in extraction buffer) alongside all historical skin extractions. The negative extraction was run along with a negative PCR control in reactions to test for contamination between samples.

### Mitochondrial DNA analysis

An 801 bp portion of the mitrochondrial gene, Cytochrome b (*cyt b*), was amplified using universal mammal primers, MVZ05 & MVZ16 [45]. We sequenced 139 *T. alpinus* and 107 *T. minimus* samples for a total dataset of 246 sequences. For the 14 historical samples, because the DNA was more degraded we were only able to amplify a fragment less than 400 bp. Therefore, we developed three pairs of genus specific primers to amplify shorter fragments that could be pieced together to complete a 780 bp sequence for the historical DNA samples (Additional file 3: Table S2). To ensure the species-specific primers were not causing any irregularities in the sequences, we also used these primers on five modern DNA samples and compared the results with the sequences using the MVZ universal primer pair. The thermal cycler conditions for the mitochondrial PCRs were as follows: 94°C for 2mins, 35 cycles of 94°C for 30s, 47-50°C for 30s, 72°C for 60s and then a final extension at 72°C for 5mins. Historical samples were sequenced in both the forward and reverse direction and PCR’d and sequenced at least twice to ensure repeatability of resulting sequence. Amplicons were sequenced on an ABI 3730 Capillary Sequencer (Applied Biosystems, Inc.). Resulting sequences were edited and aligned using Sequencher 4.8 (Gene Codes Corp.).

### Mitochondrial data analyses

We used two approaches to estimate genealogical relationships. First, to estimate the phylogenetic relationships of haplotypes, we used the Bayesian approach implemented in MRBAYES 3.1.2 [46]. The best-fit model of nucleotide change was estimated using Akaike Information Criterion as implemented in jModeltest [47]. The model of sequence evolution ranked highest by AIC for the dataset was the Tamura-Nei model (TrN + I + Γ) but because TrN is not an option in MrBayes, and this model is a special case of the general time reversible model (GTR) we used GTR + I + Γ. We ran four MCMC chains for 3,000,000 generations with trees sampled every 300 generations. We assessed convergence by examining the standard deviation of split frequencies, which were <0.01 after 3 × 10^6^ generations. A burn-in period of 10^5^ was discarded prior to calculating the consensus tree. Three individuals of the Panamint chipmunk (*Tamias panamintinus*) were used as an outgroup to *T. alpinus* and *T. minimus*[31]. Traditional phylogeny reconstruction approaches such as described above, however, make several assumptions that make them inaccurate at the population level. For example, they assume ancestral haplotypes are no longer present in the population. Therefore, our second approach was to use haplotype networks to estimate the genealogical relationship using the statistical parsimony approach [48] as implemented in the program TCS 2.1 [49].

Genetic variation among sequences within species was quantified as haplotype diversity (h_d_), and nucleotide diversity (θ_π_. θ_S_). To quantify mtDNA differentiation between species and/or populations we calculated the average and the net number of nucleotide substitutions per site (*Dxy* and *Da*, [50]). To visualize divergence patterns we clustered individuals by geography and species and used *Dxy* to produce a neighbour-joining tree in MEGA version 4 [51]. All diversity and distance calculations were estimated in program DNAsp version 5 [52]. We used an Analysis of Molecular Variance (AMOVA) implemented in the program ARLEQUIN [53] to examine the population structure of sequence diversity. F-statistic analogues (ϕ) were calculated to estimate the differentiation among groups (ϕ_CT_) among populations within groups (ϕ_SC_) and within populations (ϕ_ST_). Populations were grouped according to their species designation and sampling locality (Figure 1). We tested the statistical significance of the AMOVA with 10000 permutations and corrected the p-values associated with the ϕ values using Bonferroni correction for multiple tests. To test for historical population expansion or contraction in each species we calculated Tajima’s D [54] and Fu’s Fs statistic [55] and the 95% confidence interval around these statistics using the bootstrap method (with no recombination) offered in DNAsp [53] with 5000 replicates.

### Microsatellite analyses

We amplified the DNA at 14 microsatellite loci in *T. alpinus* and *T. minimus* (Loci names: EuAmMS26, EuAmMS37, EuAmMS41, EuAmMS86, EuAmMS94, AC A2, AC A101, AC A108, AC B12, AC B111, AC C2, AC C122, AC D107 and AC D115). The first five were taken from the literature [56], and the remaining nine were developed in *T. alpinus*. Primer sequences of all 14 loci are available in Additional file 3: Table S2. Reverse primers were fluorescently labeled with one of the following dyes: PET, NED, FAM, or HEX, forward primers were unlabeled. PCR reactions with a volume of 8.0 μl contained reagents in the following concentrations: 0.5-1 μl DNA template, 0.25 μM each primer, 0.2 mM each dNTP, 0.8 μl 10X BSA, 0.8 μl 10X PCR buffer (Roche), 1.5 mM MgCl_2_ and 0.4U of *Taq* DNA polymerase (Roche). The thermal cycler consisted of 94°C for 2 min, followed by 30 cycles of 94°C for 40s, 51-60°C for 40s, and 72°C for 40s, and ending with a final extension at 72°C for 10mins. Locus-specific annealing temperatures are shown Additional file 1: Table S1). PCR products were sized by capillary electrophoresis on an ABI 3730 sequencer (Applied Biosystems, Inc.), and alleles were scored manually using program GENEMAPPER Ver. 4.0 software (Applied Biosystems, Inc.). Positive and negative controls as well as three replicate samples were run on each PCR plate for each locus. Repeat genotypes showed high repeatability.

We tested for linkage disequilibrium and deviations from Hardy-Weinberg equilibrium (HWE) in each locus, across populations and overall with an exact test using (10000 permutations; [57]. Significant heterozygote deficiencies were used to identify the presence of null alleles as well as using the program FreeNA [58] to detect the frequencies of null alleles in our dataset. Bonferroni corrections for multiple tests were applied to p-values [59]. To examine population structure, we applied the Bayesian approach implemented in the software Structure 2.3.3 [60] to identify clusters of randomly mating individuals with minimum HW deviations and linkage disequilibrium. We ran the admixture model with correlated allele frequencies with five replicates of 10^6^ Markov Chain Monte Carlo (MCMC) iterations after a burnin of 10^5^ from K (number of parental populations) = 1 to K = 10. To provide the most accurate estimation of K, we used the statistic ∆*K* introduced by Evanno et al. [61]. We averaged coefficients of membership across the five replicates using the software CLUMMP 1.1 [62] and DISTRUCT 1.1 [63] was used to plot the graphical representation of this membership. To further examine genetic structure we used the program Arelquin [53] to calculate pair-wise F_ST_ values. To visualize the genetic distance, we generated a neighbor-joining tree using the pairwise F_ST_ distances in the program MEGA version 4 [51].

### Divergence dynamics

To infer the divergence history between *T. alpinus* and *T. minimus*, we used the coalescent-based isolation-with-migration (IM) model [64] implemented in the program IMa2 [65]. We estimated the following parameters: effective population size of *T. alpinus* (N_eALP_), *T. minimus* (N_eMIN_) and their common ancestor (N_eA_), the migration rate from *T. alpinus* into *T.minimus* (m_ALP->MIN_) and from *T. minimus* to *T. alpinus* (m_MIN->ALP_), and finally time since divergence (t). IMa2 first uses a Bayesian Markov Chain Monte Carlo (MCMC) approach to integrate over the space of possible genealogies and divergence times then uses the genealogies to estimate the posterior distribution of effective population sizes and migration rates to calculate joint posterior probability of all model parameters [65-67]. We used 10 loci (*cyt b* sequences, and 9 microsatellite loci: EuAmMS26, EuAmMS41 EuAmMS86, EuAmMS94, EuAmMS37, ACA101, ACA108, ACC2, ACD115) partitioned by species in this analysis. Five microsatellite loci used in the population genetic analyses have complex repeat motifs and therefore may not follow a strict step-wise mutation model. We sub-sampled the entire dataset to improve computational efficiency. Thirty individuals from the microsatellite dataset and twenty individuals from the sequence dataset were randomly chosen from each species for the analysis, with assurance that each geographic area was represented. We used a two-population model where each species was considered a “population”. A series of preliminary runs were used to estimate upper bounds on priors and assess mixing. Our final run consisted of 60 chains (geometric heating scheme set h_a_ = 0.980, h_b_ = 0.50), a burnin of 3 × 10^5^ steps followed by 30 × 10^6^ steps sampling trees from each locus every 300 steps (ESS > 50). We did two replicates of the final run starting with a different random number seed. Each run took approximately 72 days to finish and both returned parameter estimates that were near identical. Two hundred thousand saved genealogies (100,000 from each run) were used to calculate the joint posterior probability of the parameters in L-mode of IMa2. We used a general mammalian nucleotide substitution rate weighted across sites of 8.2 × 10^-9^[31]; see also [68]) to calculate the locus-wide mutation rate for the 801 bp segment of *cyt b* to be approximately 6.6 × 10^–6^, and the average mutation rate for the microsatellites as 1.0 × 10^-4^[69]. These mutation rates were used to convert the parameter estimates into demographic units (i.e., time in years, population size in individuals and migration rates as individuals/generation). Finally, nested models were tested to determine if the full model fit the data significantly better than models when population sizes and/or migration rates were set to equal or zero.

## Results

### Morphological data

**Figure 2:**
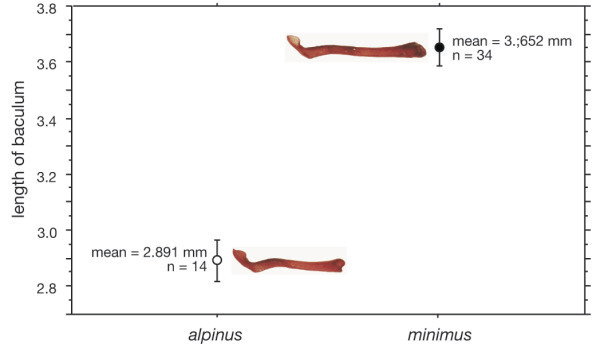
**Mean length and standard error of baculum in specimens of *T. alpinus *and *T. minimus *in Sierra Nevada, CA.** Photograph of baculum of each species shown at right of data point.

Individuals identified either as *T. alpinus* or *T. minimus* are strongly separable by bacular, external, or craniodental-mandibular characters in the multivariate canonical variates analyses of each character set (Figure 2, Additional file 4 on morphological analyses Table S3, Figure S1, S2 & S3). A few individuals of both species display intermediate posterior probabilities of group membership in external and craniodental-mandibular characters, but the number is minimal (<2% for crandiodental-mandibular characters) and not unexpected given the large number of comparisons. Importantly, there is no bias in the distribution of misclassified individuals to the geographically adjacent samples of each species (i.e., misclassified *alpinus* individuals from the T.alp-N sample are not placed in the adjacent, but non-overlapping T. min-N sample), which would be expected if there had been, or was continuing interbreeding.

### Mitochondrial sequence data

Our dataset consisted of 246 sequences with 81 variable sites, 40 singleton sites and 47 haplotypes. We found 28 haplotypes appear in *T. alpinus*, 23 haplotypes appear in *T. minimus* dataset and four shared between species (Table 1). All individual identification numbers, their locality information, and haplotype are provided in Additional file 2: Table S4. Sequences are available on GenBank (GenBank: KJ452863-KJ453106).

**Table 1:** **Sample size, number of haplotypes detected, haplotype diversity (h**_**d**_**) nucleotide diversity (θ**_**π**_**, θ**_**S**_**) and tests of population expansion/contraction (Tajima’s D, Fu’ Fs statistics) in *T. minimus *and *T. alpinus*, and geographic groups of each species (Figure **1) **at the mitochondrial gene, *cyt b***

**Species**	**N**	**No. of haplotypes**	**h**_**d**_	**θ**_**π**_	**θ**_**S**_	**Tajima’s D**	**Fu’s Fs**
*T. alpinus*	139	28	0.797	0.014	0.021	-1.581	0.094
*T. minimus*	107	23	0.891	0.016	0.012	0.932	1.624
**Geographic groups**							
T. alp-N	113	12	0.695	0.012	0.008	0.9191	7.317
T. alp-S	26	17	0.951	0.026	0.038	-1.492	-0.489
T. min-N	40	11	0.863	0.012	0.010	1.015	3.186
T. min-C	11	4	0.764	0.002	0.002	0.433	0.164
T. min- Wht/Iny	26	6	0.517	0.008	0.008	0.0976	4.225
T. min-S	30	6	0.655	0.001	0.002	-1.309	-1.697

**Figure 3:**
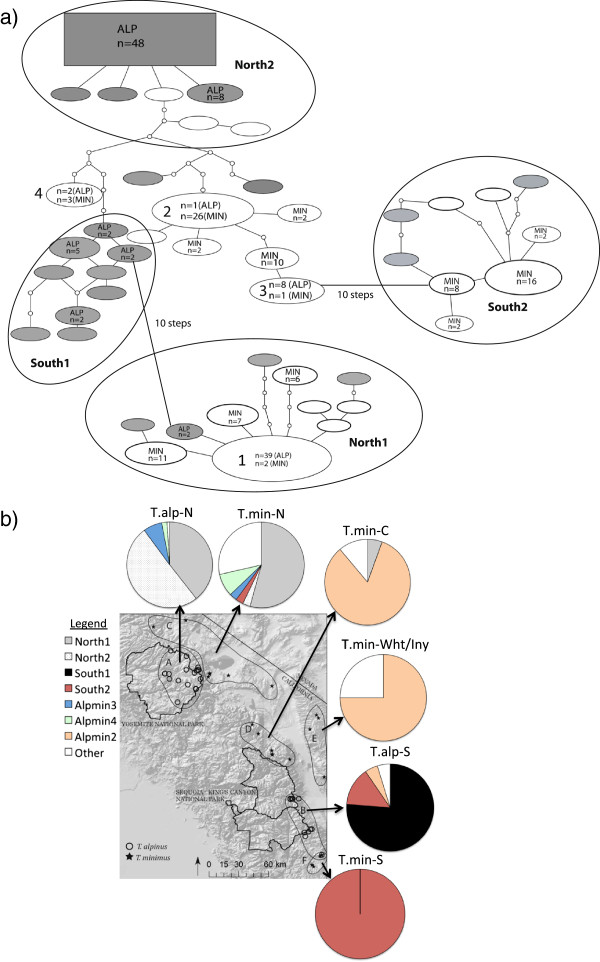
**Haplotype network and map of major haplogroups*.*a)** 95% Statistical parsimony haplotype network for *cyt b*. Haplotypes are indicated as ovals and scaled by frequency (also noted within oval unless haplotype is unique). Gray represents *T. alpinus* haplotypes, white represents *T. minimus* haplotypes and numbered shapes represent shared haplotypes 1) AlpMin1, 2) AlpMin2, 3) AlpMin3, and 4) AlpMin4. Large ovals show groups of individuals with similar haplotypes (“haplogroups”) from the same geographic region. There are two distinct northern haplogroups (North1 and North2) and two distinct southern haplogroups (South1 and South2). If haplotypes are not encompassed within an oval, no geographic pattern exists (i.e, found throughout sampling area); **b)** Pie charts showing the percentage of individuals from each geographic sampling group belonging to the major haplogroups and shared haplotypes (Alpmin 2, 3, & 4; note that Alpmin1 is within Haplogroup North1).

There were four haplotypes that were shared by both species. Three shared haplotypes occurred between the T.alp-N and T.min-N sampling localities and one was shared between T.alp-S, T.min-Wht/Inyo and T. min-C groups (Figure 3). The first shared haplotype (AlpMin1) was the second most frequent haplotype present in *T. alpinus* (29% of all individuals) where it was confined to individuals from the Yosemite area (T.alp-N) and two from the northern sampling area of *T. minimus* (T. min-N; Figure 3; Additional file 5: Figure S4). AlpMin1 was the most frequent haplotype found in a northern haplogroup we named “North1”. The next shared haplotype (AlpMin2) was the most frequent haplotype found in *T. minimus* (24%) and detected in only one *T. alpinus* individual from Bullfrog Lake (T. alp-S). All *T. minimus* individuals with the AlpMin2 haplotype were from the White Mountains, the Inyo Mountains or the central part of our sampling area (Figure 3; T. min-Wht/Iny & T.min-C). Interestingly, this was the only haplotype that was shared between southern *T. alpinus* individuals and any of the *T. minimus* groups. The third shared haplotype (AlpMin3) was in low frequency (6%) and only found at one site in Yosemite National Park in *T. alpinus* (Vogelsang Lake, T. alp–N) and in one *T. minimus* sampled nearby at Bohler Creek (T. min–N). The fourth and last shared haplotype (AlpMin4) was also in low frequency in both species (*T. alpinus*: 1.4%; *T. minimus*: 2.8%) from the northern part of our sampling area. The geographic pattern of shared haplotypes is consistent with historical hybridization events in both the northern and southern portion of *T.alpinus’* range.

The statistical parsimony haplotype network for *T. alpinus* and *T. minimus* had a 95% parsimony limit of 12 steps (Figure 3a). The mtDNA phylogenetic tree estimated by the Bayesian analysis was weakly resolved but supports the network analysis and demonstrates a lack of clear genealogical separation between the two species (Additional file 5: Figure S4). There are four groups of haplotypes that are separated by at least 5 base pair changes and show some geographic structure (Haplogroups North1, North2, South1 and South2, Figure 3b) and three out of four of these contain individuals of both species (North1, North2 and South2). South1 is made up of only *T. alpinus* individuals from the southern portion of their range. A map of the haplogroups and shared haplotypes shows that genetic similarity is more defined by geography than by species identity (Figure 3b). Two southern *T. alpinus* haplotypes were more than 12 steps away from the others in the network and are not shown in Figure 3.

The average and the net number of nucleotide substitutions per site were lower between species (Dxy = 0.018, Da = 0.003) than between the northern and southern *T. alpinus* populations (Dxy = 0.021, Da = 0.005). The northern *T. alpinus* population was most similar to the northern *T. minimus* population (Dxy = 0.015, Da = 0.003, Table 2; Additional file 6: Figure S5). The southern *T. minimus* samples are the most genetically distinct group sampled, being most different from the northern *T. minimus* population (Dxy = 0.024, Da = 0.017; Table 2, Additional file 6: Figure S5). There were no significant signals of population expansion or decline (or deviations from neutrality) in either species or geographic populations of species according to Tajima’s D or Fu’s F_s_ statistics in any of the groups tested (Table 1).

**Table 2:** **Pairwise comparisons of *Tamias *** **populations**

	**T. alp-N**	**T. alp-S**	**T. min-N**	**T. min-C**	**T. min-Wht/Iny**	**T. min-S**
T. alp-N	-	0.020	0.015	0.017	0.016	0.023
T. alp-S	**0.116**	-	0.022	0.020	0.021	0.024
T. min-N	**0.167**	**0.092**	-	0.017	0.015	0.024
T. min-C	**0.166**	**0.103**	**0.018**	-	0.005	0.019
T. min-Wht/Iny	**0.167**	**0.114**	**0.028**	**0.042**	-	0.020
T. min-S	**0.227**	**0.168**	**0.118**	**0.124**	**0.147**	-

Statistically significant Fst values shown in bold font (P < 0.005, 10000 permutations).Pairwise Dxy values from the mtDNA data are given above the diagonal and pairwise Fst-values from the microsatellite data below the diagonal.

Consistent with the above, the AMOVA attributed 28.63% of the genetic variation across haplotypes to be between species (ϕ_CT_ = 0.28, p = 0.34), 37.6% to be among populations within species (ϕ_SC_ = 0.52, p < 0.001) and 33.8% of the variation to differences within species (ϕ_ST_ = 0.66, p < 0.0001). By comparison, the AMOVA for the analysis of nuclear loci (see next section) attributed 62% to variation among groups (ϕ_CT_ = 0.62, p = 0.14), 5.1% among populations within groups (ϕ_SC_ = 0.13, p < 0.001) and 32.9% to variation within populations (ϕ_ST_ = 0.67, p < 0.001). The AMOVA analyses reveal that mtDNA variation is not explained by differences among species or geographic groups, but rather differences within species and populations.

### Microsatellite data

Deviations from H-W equilibrium were observed across all loci within species however this is not unexpected given the observed genetic substructure. Within geographic groups, there were deviations from HWE in the *T. alpinus* N group but again, there is known substructure in this group [70] so deviations from HWE are expected. A high frequency null allele was detected at the D107 locus in the *T. alpinus*. We ran the STRUCTURE analysis with and without this locus and the results did not change, therefore, we chose to run the analysis with all 14 loci. No significant linkage disequilibrium was detected after Bonferonni correction. Genetic diversity was highest in the T. min-N group (*A =* 7.6; H_e_ = 0.84), followed by T. min –Wht/Iny (*A =* 6.8; H_e_ = 0.84; Table 3). The northern *T. alpinus* samples had the lowest genetic diversity of all sampled groups (*A =* 4.7, H = 0.63). Microsatellite genotype data is available in Additional file 7.

**Table 3:** **Sample size (N), average allelic richness (*A*; corrected for differences in sample size), observed and expected heterozygosity (H**_**o**_**, H**_**e**_ &**standard deviation (sd)) in *T. alpinus *and *T. minimus *at 14 microsatellite loci**

**Species**	**Locality**	**N**	***A***	**H**_**o**_**(sd)**	**H**_**e**_**(sd)**
*T. alpinus*	North (YNP)	149	4.7	0.57 (0.19)	0.63 (0.21)
	South	17	6.0	0.71 (0.19)	0.75 (0.17)
*T. minimus*	North	57	7.6	0.73 (0.14)	0.84 (0.07)
	White/inyo mtns	33	6.8	0.71 (0.18)	0.82 (0.09
	Central	42	6.4	0.62 (0.12)	0.78 (0.10)
	South	29	5.1	0.58 (0.23)	0.68 (I0.18)

### Population structure

**Figure 4:**
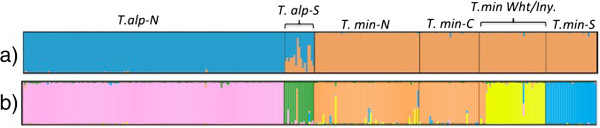
**Bayesian analysis of nuclear genetic structure of *Tamias *populations based on 14 microsatellite loci.** Each individual is represented by a vertical line, which is partitioned into colored segments that indicate individual’s membership in **(a)** 2 or **(b)** 5 parental populations.

The estimated number of parental populations for the microsatellite dataset using the Evanno method was K = 2, however the mean likelihood values were higher at K = 5 and above. The results of the cluster analyses at K = 2 separated individuals into two groups by species, with admixture in the T. alp-S (Figure 4a). However, the pairwise F_ST_ values between T.alp-S and T.alp-N, and T.alp-S and all of the *T. minimus* sampling localities show significant genetic differentiation (e.g., pairwise F_ST_ =0.116 between T.alp-N & T.alp-S and 0.092 between T.min-N & T.alp-S, p < 0.005, see Table 2), suggesting that hybridization is not ongoing. There was a higher mean likelihood value at K = 5 and above, than at K = 2, also indicating that K = 5 more likely exhibits the genetic structure of these two species across our study area (Additional file 8; Figure 4b). The individual membership graph for K = 5 shows further geographic subdivision within *T. minimus* (Figure 4b), with T.alp-S highly differentiated from T.alp-N. Based on cluster membership percentages, T. min-N & T.min-C is one genetic cluster showing evidence of gene flow with T.min-Wht/Iny, while T.min-S is well differentiated. (Additional file 8, Figure 4a & b). K values above five did not greatly improve likelihood scores but do reveal subdivision between T.min-N & T.min-C and within T.alp-N (graphs not shown). The T.alp-S sample appears as a distinct cluster, with one exception. There is one *T. alpinus* individual from Bullfrog Lake (MVZ224480) in the T.alp-S geographic group that was assigned to T.Min-N based on its genotype at 14 loci (Figure 4b). This individual had a divergent mtDNA haplotype shown with arrow on Bayesian tree (location shown with green triangle on the map of Additional file 5: Figure S4) and morphologically is unambiguously identified as a *T. alpinus*.

The pairwise F_ST_ values between clusters showed significant differentiation across all clusters and ranged from 0.018 between T.min-N and T.min-C to 0.227 between T.alp-N and T.min-S (Table 2). In contrast to the results for mtDNA, the neighbor-joining tree based on F_ST_ shows that groups of the same species are genetically more similar to each other than to the other species (Additional file 8: Figure S7) although the southern populations of both species (T. alp-S and T.min-S) are both differentiated from their more northern conspecifics.

### Coalescent analysis of divergence history

**Figure 5:**
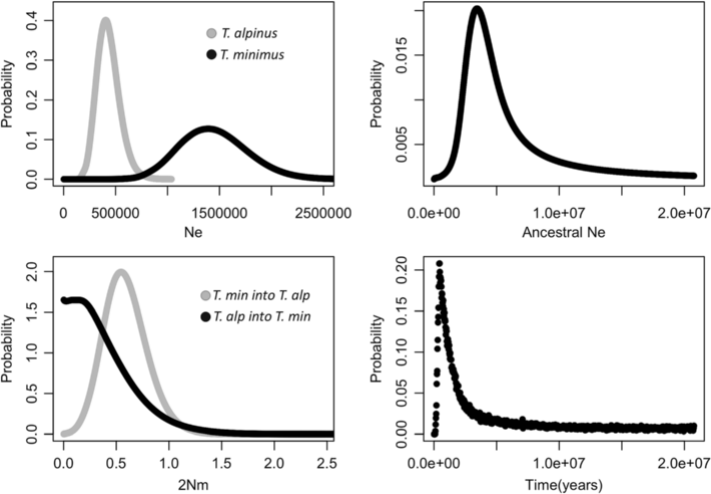
**Posterior density plots of parameters from Isolation with Migration (IMa2) analysis.** Top left: effective population size of *T. alpinus* and *T. minimus*; top right: ancestral effective population size; bottom left: effective number of migration events per generation between *T. alpinus* and *T. minimus*; bottom right: divergence time between *T. alpinus* and *T. minimus*.

Using IMa2, we estimated the following parameters: effective population size of *T. alpinus* (Ne_ALP_), *T. minimus* (Ne_MIN_) and the common ancestor (Ne_A_) the migration rate from *T. alpinus* into *T.minimus* (m_ALP->MIN_) and from *T. minimus* to *T. alpinus* (m_MIN->ALP_) and time since divergence (t) (Table 4). The split between *T. alpinus* and *T. minimus* lineages was estimated by IMa2 to have occurred in the mid-Pleistocene, at approximately 450 ka. There is a sharp peak in the posterior density plot at this value however, the plot plateaus at a low, but non- zero value for higher values of t, including when a higher upper bound on the divergence time prior is used (results not shown). The mean effective population size (N_e_) of *T. alpinus* was estimated to be much smaller than *T. minimus* with non-overlapping confidence intervals (T. alp mean N_e_ = 430,625. 95% HPD 230,019- 648,519; T. min mean N_e_ = 1,448,317, 95% HPD 833,365-2,095,096). The size of the daughter populations is small compared to the ancestral population (N_eA_ = 6,680,761). Migration estimates between the two species showed strong evidence for unidirectional migration from *T. minimus* into *T. alpinus* (2*N*_m_ = 0.5441, p < 0.001). Migration from *T. alpinus* into *T. minimus* (2 *N*m = 0.002) was not significantly different from zero (Table 4; Figure 5). Testing of 24 nested models using the Likelihood Ratio Test (LRT) rejected models with zero migration rates and the nested model with the strongest support allowed migration from *T. minimus* into *T. alpinus* but zero migration in the other direction. This nested model had the same log-likelihood as the full model, and had the highest log likelihood out of all 24 models tested, providing strong support that the unidirectional migration model is a good description of the data.

**Table 4:** **Parameter estimates from IMa2 runs for *T. alpinus *** **and *T. minimus***

	**N**_**e**_	**N**_**eA**_	**t**	**2N**_**m **_**T.min to T. alp**	**2N**_**m **_**T.alp to T.min**
*T. alpinus*	430625	6680761	446538	**0.5441**	0.002
HPD95 low-high	230019-648513	778846-18058846*	115 -*	0.1986-0.9981*	0-0.9463*
*T. minimus*	1448317				
HPD95 low-high	833365-2095096				

*HPD may not be useful because posterior density does not reach low levels near upper (N_eA_, t) or lower (2 *N*m) limit of prior. In the case of N_eA_ & t, even with higher values set for the prior, the posterior density plot plateaus at low but nonzero values (see Figure 5).

Bold font shows significance of *p <* 0.001*.*N_e_ estimates are mean values and migration rate parameters are the HiPt of the posterior probability.

## Discussion

We examined the evolutionary relationship of *T. alpinus* and *T. minimus* using cytochrome b and microsatellites to help elucidate the divergence history of *T. alpinus* in the Sierra Nevada. Microsatellite analyses were used to provide a contemporary view of this relationship and to examine details of population genetic structure within and across species. We found that *T. alpinus* and *T. minimus* populations share mitochondrial haplotypes with no overall geneaological separation, and that diversity at this locus is better explained by geography than by species’ boundaries. This pattern indicates either recent speciation of *T. alpinus* from *T. minimus* with retention of ancestral polymorphism, or extensive introgression subsequent to splitting. In contrast to the mtDNA sequence data, the analyses of nuclear microsatellite loci and morphology revealed that the two species are genetically distinct. Although there are highly differentiated populations within species, populations of the same species are more similar to each other than they are to members of the other species. This suggests that contemporary hybridization is not widespread along the geographic boundary between *T. minimus* and *T. alpinus.* Coalescent analysis of divergence history revealed mid to late Pleistocene divergence and low, but significant gene flow between the two lineages. Overall, our study suggests that: speciation between *T. alpinus* and *T. minimus* is relatively recent, secondary contact and historical introgression has occurred, and there is little evidence or opportunity for contemporary hybridization given the current distribution of these two species.

Differentiating between the genetic signature of incomplete lineage sorting and historical hybridization is difficult; however, distinguishing between the two is important in addressing non-concordance among characters in closely related species [71]. The spatial pattern of genetic variation across species can help to provide an objective assessment of which process is more likely to have occurred because each should produce a specific spatial pattern [30,72]. The results of our mitochondrial sequences show no strong spatial structure within species. Both the Bayesian tree and haplotype network indicate the two species are completely intermingled across the landscape. Recent hybridization should show a clustered pattern, where introgressed alleles are more common at or near the contact zone of the two species; in contrast, ancestral polymorphism should be diffuse and uniform across space [30]. However, the spatial patterns described above assume a contact zone between species, and for this study, we were unable to obtain samples of both species from the same location. The one site where they were collected in close proximity in 1911 (Little Brush Meadow, Tulare Co., CA) showed distinct and divergent haplotypes between species. The single *T. minimus* taken from this locality had a unique haplotype that clustered with the southern *minimus* haplogroup in the network (South2 Figure 3, Additional file 5: Figure S4) whereas the *T. alpinus* from that location and three other *T. alpinus* from the general area collected at the same time, all clustered within southern *T. alpinus* haplogroup (South1). Overall, the distribution of shared haplotypes reflects geographic proximity, not current contact; northern *T. alpinus* and *T. minmus* shared three haplotypes and southern *T. alpinus* and central and southern *T. minimus* shared one. This geographic pattern, along with the results of the IMa2 analysis suggest that hybridization occurred at some point in the past but given the results of the microsatellite analysis and current distribution of the two species, hybridization is not currently ongoing.

There were potential problems with the parameter estimates using IMa2, in particular, that for divergence time. The first is that our microsatellite data exhibit genetic structure within lineages (recall that in this analysis, each species was considered a “population”), which may lead to an overestimation of divergence time [73]. Second, the right tail of the posterior density plot reached low but non-zero values potentially making the 95% HPD high and low estimates unreliable (i.e. posterior density distribution did not converge within prior range). Given these caveats, the estimate of divergence time between *T. alpinus* and *T. minimus* from IMa2 is about 450 ka with some support of it being as recent as 110 ka (the lower 95% HPD of t). This timeframe occurs in the mid to late Pleistocene, a time of extreme climate fluctuations in the Sierra Nevada including several major glaciations with prolonged glacial rather than interglacial periods [9]. The coalescent analysis also supports low but significant gene flow subsequent to splitting from *T. minimus* into *T. alpinus*. Given the relatively small population size estimated by the IMa2 analysis for *T. alpinus* compared to *T. minimus* and considering that *T. alpinus* is a range restricted endemic, it is conceivable that the *T. alpinus* population would receive more genes from *T. minimus* than the other way around.

The Pleistocene shaped the genetic structure and distributions of many species (reviewed in [7]) and its role in the speciation in several North American taxa, though sometimes debated, is clear (e.g., [74-76]). It is plausible that a founding population of *T. alpinus* became isolated in a refugium from an adjacent *T. minimus* population. A recent study that used sequence data from reproductive protein genes showed that *T. alpinus*, characterized by strongly divergent sequences*,* is a monophyletic group nested within *T. minimus* species complex [32]. This nested pattern is consistent with expectations for a peripheral isolate of a widespread species providing further support to our results suggesting that speciation of *T. alpinus* is recent*.* The IMa2 analysis also supports this notion with a parameter estimate for the effective population size of *T. alpinus* (N_eALP_ = 430,625), substantially smaller than the estimate of the effective population size of *T. minimus* (N_eMIN_ = 1,448,317). Finally, although the estimates of migration between species were low (95% HPD high <1 from *T. minimus* to *T. alpinus*), there is significant evidence of historical genetic introgression between species, adding to the complexity of the divergence dynamics between these two species. The results of this study adds to the growing number of studies that have highlighted the importance of glacial-interglacial refugia in recently derived species across taxa in the central and southern Sierra Nevada (e.g., [12,14]).

Hybridization can play an important role in the evolution of species [77-79]. It was previously accepted that the morphological differences in bacular morphology in western chipmunks mechanically prevented hybridization between species and was considered a strong pre-mating barrier to gene flow [43,80]. However, several recent studies have documented both historical and ongoing hybridization in two non-sister species of *Tamias* and suggest that it may be more common in the genus than previously thought [30,31,42]. Our analyses provide little or no evidence for current introgression across the species boundaries; however, the potential for contemporary gene flow between these species where they come into close proximity in the southern portion of their range exists. One apparent hybrid individual, morphologically determined to be a *T. alpinus* with a divergent *T. alpinus* haplotype, was more similar to *T. minimus* than *T. alpinus* based on 14 microsatellite loci. The assignment of this individual to the T.min-N population is puzzling because the two localities are far apart geographically (at least 80 km). Despite extensive survey effort along the eastern flank of the Sierra Nevada, we have not found a locality of co-occurrence where there is direct potential for hybrid matings. Furthermore, our morphological analyses show no evidence of hybridization and species were easily discriminated based on the measured morphological characters.

## Conclusions

Speciation is a prolonged process that likely has phases in different spatial contexts [81,82]. Here, we provided evidence of recent speciation with limited yet significant post-divergence gene flow between the Alpine chipmunk and its closest relative. Our approach revealed an interesting and complex pattern of shared and intermingled haplotypes across species and highly differentiated populations within. The presence of geographic structure of shared haplotypes between these closely related lineages could be a result of allopatric speciation with secondary contact, but the same pattern could be a result of zones of primary parapatric speciation. As theory suggests, any mechanism that can cause divergence in allopatry can also occur in parapatry, as long as the selective gradient acting on differentiation is strong enough to counterbalance continuous gene flow [83]. In order to further examine the genetic patterns that emerged in this study, future work comparing genome-level patterns of diversity between these two species may reveal regions of the genome under strong divergent selection; a pattern not expected in an allopatric speciation scenario [84]. Additionally, environmental niche models (ENMs) used to predict historical ranges aid in the identification of areas of isolation and the potential for secondary contact in the past. The environmental modeling results can be combined with genetic data to test alternative hypotheses of allopatric speciation with secondary contact or primary parapatric speciation (e.g. [85]). A multifaceted approach including building historical ENMs and genomic data combined with increased sampling could further improve our understanding of the evolutionary history of Alpine chipmunk, a species that appears to be under threat due to recent climate change [70,86]. A clear understanding of this species evolutionary history could help us understand its vulnerability in the face of environmental change.

## Competing interests

The authors declare that they have no competing interest.

## Authors’ contributions

EMR and JLP collected the samples, with assistance from members of the Grinnell Resurvey Project field team. EMR carried out the lab work, data analysis and interpretation, and wrote the manuscript. JLP carried out the morphological analyses. JLP and CM provide the resources and context for the study, discussed the design and general methods of analysis and edited the manuscript. EMR, JLP and CM all read and approved the final manuscript.

## Supplementary Material

Additional file 1: Table S1 Summary of species-specific differences between *T. alpinus* and *T. minimus.* Click here for file

Additional file 2: Table S4 ID/Catalogue numbers of all samples sequenced at *cytochrome b.* Click here for file

Additional file 3: Table S2 Microsatellite and *cytochrome b* primers used in analyses. Click here for file

Additional file 4 Detailed results and additional figures for morphological analyses. Click here for file

Additional file 5: Figure S4 Bayesian estimate (GTR + I + Γ) of phylogeny of *cyt b*. Click here for file

Additional file 6: Figure S5 NJ tree of the relationships among geographic groups of *T. alpinus* and *T. minimus* based on the average number of nucleotide substitutions per site (Dxy, Nei [51]) at *cyt b*. Click here for file

Additional file 7 Microsatellite Genotype Data used in STRUCTURE analysis. Click here for file

Additional file 8: Figure S6 Estimation of the true number of clusters using ∆K (Evanno et al [62]) and Figure S7. Unrooted NJ tree of the relationship among populations using F_ST_. Click here for file
