# Supplementary material for: Diversification of the Alpine Chipmunk, Tamias alpinus, an alpine endemic of the Sierra Nevada, California
Source: BMC Evol Biol. 2014 Feb 23;14:34. doi: 10.1186/1471-2148-14-34 (PMC4077034; doi:10.1186/1471-2148-14-34)
Supplement: Additional file 1: Table S1 — Summary of species-specific differences between T. alpinus and T. minimus. [file 1471-2148-14-34-S1.pdf]

Supplementary Information

Recent speciation or reticulate evolution? Diversification of the Alpine Chipmunk, *Tamias alpinus,* an alpine endemic of the Sierra Nevada, California.

Emily M. Rubidge, James L. Patton and Craig Moritz

Table S1. Summary of species-specific differences between *T. alpinus* and *T. minimus scrutator (*for the purposes of this study *T. m. scrutator* is referred to as *T. minimus)*

Species-specific character	Known difference
Color and color pattern	*T. alpinus* is dominated by pale yellow tones overall with a distinctly indistinct lateral strip, which contrasts with the overall gray tones of *T. minimus* with equally intensely brown stripes, including the most lateral one
Tail length and bushiness	*T. alpinus* has a shorter but bushier tail than *T. minimus*, which has a noticeably narrow, elongated tail.
Ear length	*T. alpinus* has longer ears, ranging from 16-18 mm; *T. minimus* shorter ears, ranging from 14-16 mm.
Baculum (penis bone)	*T. alpinus* bacula are distinctly shorter with a broader tip than *T. minimus*
Behavior	*T. alpinus* runs with its tail held horizontally, undulating frequently; when stationary, the tail is held relatively still, with the tip not flicking up and down. By contrast, *T. minimus* runs across open space with the tail held stiffly vertically, never undulating, and the tail flips up and down constantly when an individual is sitting
Habitat	Alpine chipmunks are restricted to the upper belt of 5-needle pines (white-bark pine, *Pinus albicaulis*), foxtail pine, *Pinus balfouriana*) and the open scree habitats above timberline, at elevations largely above 10,000 ft (3,050 m). The Least chipmunk has a wide elevational amplitude, ranging in eastern California from about 6,500 ft (1,980 m) to above timberline at elevations equivalent to those of *T. alpinus*. *T. minimus* however, is largely limited to open sagebrush habitats, which may span this entire elevational range in the central Sierra Nevada and higher mountains to the immediate east (e.g., White Mts., which reach elevations above 14,000 ft [4,270 m]).

References: Sutton 1992 and Verts and Carraway 2001
