# Supplementary material for: Diversification of the Alpine Chipmunk, Tamias alpinus, an alpine endemic of the Sierra Nevada, California
Source: BMC Evol Biol. 2014 Feb 23;14:34. doi: 10.1186/1471-2148-14-34 (PMC4077034; doi:10.1186/1471-2148-14-34)
Supplement: Additional file 2: Table S4 — ID/Catalogue numbers of all samples sequenced at cytochrome b. [file 1471-2148-14-34-S2.pdf]

Table S4. ID/Catalogue numbers of all samples sequenced at cytochrome b. Summarized information includes location information, haplotype number, network group, clade and whether or not sample was included in microsatellite analysis. Samples from museum skins were not run with the microsatellites because only a subset of microsatellite (7) amplify the historical samples.

Cat. No.	Species	Location	Lattitude	Longnitude	GeogGrp	Msat	Haplotype	Network Gp	Clade
14931	*T. alpinus*	W slope Olancha Peak, Sierra Nevada	36.26623	-118.11771	T.alp_S	No	Hap_16	South1	RED
14932	*T. alpinus*	W slope Olancha Peak, Sierra Nevada	36.26623	-118.11771	T.alp_S	No	Hap_17	South1	RED
14933	*T. alpinus*	W slope Olancha Peak, Sierra Nevada	36.26623	-118.11771	T.alp_S	No	Hap_18	South1	RED
224075	*T. alpinus*	Muir Lake, Cottonwood Lakes Basin	36.49873	-118.2077	T.alp_S	Yes	Hap_28	South2	RED
224076	*T. alpinus*	Muir Lake, Cottonwood Lakes Basin	36.49873	-118.2077	T.alp_S	Yes	Hap_29	N/A	BLACK
224077	*T. alpinus*	Muir Lake, Cottonwood Lakes Basin	36.49873	-118.2077	T.alp_S	Yes	Hap_21	South1	RED
224078	*T. alpinus*	Muir Lake, Cottonwood Lakes Basin	36.49873	-118.2077	T.alp_S	Yes	Hap_21	South1	RED
14973	*T. alpinus*	Cottonwood Lakes, Sierra Nevada Mts.	36.49835	-118.21991	T.alp_S	No	Hap_17	South1	RED
14903	*T. alpinus*	Cirque Peak, Sierra Nevada	36.47629	-118.23710	T.alp_S	No	Hap_13	South1	RED
14904	*T. alpinus*	Cirque Peak, Sierra Nevada	36.47629	-118.23710	T.alp_S	No	Hap_13	South1	RED
14908	*T. alpinus*	Whitney Meadow, Sierra Nevada	36.43413	-118.26710	T.alp_S	No	Hap_14	South1	RED
14909	*T. alpinus*	Whitney Meadow, Sierra Nevada	36.43413	-118.26710	T.alp_S	No	Hap_15	South2	RED
206401	*T. alpinus*	Siberian Outpost, Sequoia National Park	36.46957	-118.2737	T.alp_S	Yes	Hap_22	South1	RED
206403	*T. alpinus*	Siberian Outpost, Sequoia National Park	36.4685	-118.2745	T.alp_S	Yes	Hap_24	South1	RED
206402	*T. alpinus*	Siberian Outpost, Sequoia National Park	36.46895	-118.2746	T.alp_S	Yes	Hap_23	South2	RED
206400	*T. alpinus*	Siberian Outpost, Sequoia National Park	36.46829	-118.2748	T.alp_S	Yes	Hap_21	South1	RED
17576	*T. alpinus*	Kearsarge Pass, Onion Valley, Sierra Nevada	36.77450	-118.32419	T.alp_S	No	Hap_19	South1	RED
206398	*T. alpinus*	Kearsarge Lakes, Kings Canyon National Park	36.77289	-118.379	T.alp_S	Yes	Hap_20	N/A	GREEN
206397	*T. alpinus*	Kearsarge Lakes, Kings Canyon National Park	36.7724	-118.3834	T.alp_S	Yes	Hap_21	South1	RED
206396	*T. alpinus*	Kearsarge Lakes, Kings Canyon National Park	36.7713	-118.3857	T.alp_S	Yes	Hap_20	N/A	GREEN
206399	*T. alpinus*	Kearsarge Lakes, Kings Canyon National Park	36.77128	-118.386	T.alp_S	Yes	Hap_18	South1	RED
224480	*T. alpinus*	Bullfrog Lake, Kings Canyon National Park	36.769664	-118.4018	T.alp_S	Yes	Hap_20	N/A	GREEN
224481	*T. alpinus*	Bullfrog Lake, Kings Canyon National Park	36.769664	-118.4018	T.alp_S	Yes	Hap_21	South1	RED
224482	*T. alpinus*	Bullfrog Lake, Kings Canyon National Park	36.769664	-118.4018	T.alp_S	Yes	Hap_25	N/A	GREEN
224483	*T. alpinus*	Bullfrog Lake, Kings Canyon National Park	36.773289	-118.4028	T.alp_S	Yes	Hap_26	-	RED
224484	*T. alpinus*	Bullfrog Lake, Kings Canyon National Park	36.773289	-118.4028	T.alp_S	Yes	Hap_27*	-	RED
222200	*T. alpinus*	Mono Pass	37.85461	-119.2136	T.alp-N	Yes	Hap_1	North2	RED
222203	*T. alpinus*	Mono Pass	37.85461	-119.2136	T.alp-N	Yes	Hap_7*	-	RED
JLP24301	*T. alpinus*	Mono Pass	37.85461	-119.2136	T.alp-N	Yes	Hap_1	North2	RED
JLP24302	*T. alpinus*	Mono Pass	37.85461	-119.2136	T.alp-N	Yes	Hap_1	North2	RED
JLP24310	*T. alpinus*	Mono Pass	37.85461	-119.2136	T.alp-N	Yes	Hap_1	North2	RED
T174	*T. alpinus*	Mono Pass	37.85461	-119.2136	T.alp-N	Yes	Hap_1	North2	RED
T175	*T. alpinus*	Mono Pass	37.85461	-119.2136	T.alp-N	Yes	Hap_1	North2	RED
T180	*T. alpinus*	Mono Pass	37.85461	-119.2136	T.alp-N	Yes	Hap_1	North2	RED

Cat. No.	Species	Location	Lattitude	Longnitude	GeogGrp	Msat	Haplotype	Network Gp	Clade
T184	*T. alpinus*	Mono Pass	37.85461	-119.2136	T.alp-N	Yes	Hap_1	North2	RED
T191	*T. alpinus*	Mono Pass	37.85461	-119.2136	T.alp-N	Yes	Hap_3*	North1	BLUE
T195	*T. alpinus*	Mono Pass	37.85461	-119.2136	T.alp-N	Yes	Hap_1	North2	RED
T196a	*T. alpinus*	Mono Pass	37.85461	-119.2136	T.alp-N	Yes	Hap_1	North2	RED
T196b	*T. alpinus*	Mono Pass	37.85461	-119.2136	T.alp-N	Yes	Hap_1	North2	RED
T197a	*T. alpinus*	Mono Pass	37.85461	-119.2136	T.alp-N	Yes	Hap_1	North2	RED
T197b	*T. alpinus*	Mono Pass	37.85461	-119.2136	T.alp-N	Yes	Hap_1	North2	RED
T198	*T. alpinus*	Mono Pass	37.85461	-119.2136	T.alp-N	Yes	Hap_3*	North1	BLUE
T38	*T. alpinus*	Mono Pass	37.85461	-119.2136	T.alp-N	Yes	Hap_1	North2	RED
EMR91	*T. alpinus*	Head of Ellery Lake	37.93635	-119.22753	T.alp-N	Yes	Hap_1	North2	RED
EMR90	*T. alpinus*	Head of Ellery Lake	37.93557	-119.22878	T.alp-N	Yes	Hap_3*	North1	BLUE
23335	*T. alpinus*	Warren Fork of Lee Vining Creek	37.96006	-119.23465	T.alp-N	No	Hap_10	North2	RED
23334	*T. alpinus*	near Ellery Lake, Tioga Rd.	37.93806	-119.23593	T.alp-N	No	Hap_3*	North1	BLUE
23338	*T. alpinus*	near Tioga Rd.	37.93248	-119.24846	T.alp-N	No	Hap_11	North1	BLUE
217179	*T. alpinus*	Upper Lyell Canyon, YNP	37.76458	-119.2521	T.alp-N	Yes	Hap_1	North2	RED
217180	*T. alpinus*	Upper Lyell Canyon, YNP	37.76458	-119.2521	T.alp-N	Yes	Hap_1	North2	RED
217181	*T. alpinus*	Upper Lyell Canyon, YNP	37.76458	-119.2521	T.alp-N	Yes	Hap_1	North2	RED
217182	*T. alpinus*	Upper Lyell Canyon, YNP	37.76458	-119.2521	T.alp-N	Yes	Hap_2	North1	BLUE
217183	*T. alpinus*	Upper Lyell Canyon, YNP	37.76458	-119.2521	T.alp-N	Yes	Hap_1	North2	RED
217184	*T. alpinus*	Upper Lyell Canyon, YNP	37.76458	-119.2521	T.alp-N	Yes	Hap_1	North2	RED
217185	*T. alpinus*	Upper Lyell Canyon, YNP	37.76458	-119.2521	T.alp-N	Yes	Hap_1	North2	RED
EMR37	*T. alpinus*	Upper Lyell Canyon , YNP	37.76458	-119.25213	T.alp-N	Yes	Hap_3*	North1	BLUE
EMR32	*T. alpinus*	Upper Lyell Canyon, YNP	37.76595	-119.25273	T.alp-N	Yes	Hap_2	North1	BLUE
EMR38	*T. alpinus*	Upper Lyell Canyon, YNP	37.76612	-119.25287	T.alp-N	Yes	Hap_4	North1	RED
EMR33	*T. alpinus*	Upper Lyell Canyon, YNP	37.76405	-119.25298	T.alp-N	Yes	Hap_1	North2	RED
216270	*T. alpinus*	Upper Lyell Canyon, YNP	37.76807	-119.2551	T.alp-N	Yes	Hap_3*	North1	BLUE
217178	*T. alpinus*	Upper Lyell Canyon, YNP	37.76807	-119.2551	T.alp-N	Yes	Hap_1	North2	RED
217186	*T. alpinus*	Upper Lyell Canyon, YNP	37.76807	-119.2551	T.alp-N	Yes	Hap_1	North2	RED
EMR36	*T. alpinus*	Upper Lyell Canyon, YNP	37.76417	-119.25540	T.alp-N	Yes	Hap_3*	North1	BLUE
EMR35	*T. alpinus*	Upper Lyell Canyon, YNP	37.76415	-119.25542	T.alp-N	Yes	Hap_1	North2	RED
EMR31	*T. alpinus*	Upper Lyell Canyon, YNP	37.76397	-119.25582	T.alp-N	Yes	Hap_1	North2	RED
EMR34	*T. alpinus*	Upper Lyell Canyon, YNP	37.76390	-119.25598	T.alp-N	Yes	Hap_1	North2	RED
EMR29	*T. alpinus*	Upper Lyell Canyon, YNP	37.76353	-119.25625	T.alp-N	Yes	Hap_1	North2	RED
EMR30	*T. alpinus*	Upper Lyell Canyon, YNP	37.76353	-119.25625	T.alp-N	Yes	Hap_1	North2	RED
EMR50	*T. alpinus*	Upper Lyell Canyon, YNP	37.76330	-119.25773	T.alp-N	Yes	Hap_3*	North1	BLUE
EMR39	*T. alpinus*	Upper Lyell Canyon, YNP	37.76228	-119.25847	T.alp-N	Yes	Hap_3*	North1	BLUE
217188	*T. alpinus*	Upper Lyell Canyon, YNP	37.75675	-119.2586	T.alp-N	Yes	Hap_8	North2	RED
EMR48	*T. alpinus*	Upper Lyell Canyon, YNP	37.76150	-119.25870	T.alp-N	Yes	Hap_1	North2	RED
EMR51	*T. alpinus*	Upper Lyell Canyon, YNP	37.76155	-119.25885	T.alp-N	Yes	Hap_1	North2	RED
EMR41	*T. alpinus*	Upper Lyell Canyon, YNP	37.76072	-119.25923	T.alp-N	Yes	Hap_3*	North1	BLUE
EMR45	*T. alpinus*	Upper Lyell Canyon , YNP	37.76072	-119.25923	T.alp-N	Yes	Hap_3*	North1	BLUE

Cat. No.	Species	Location	Lattitude	Longnitude	GeogGrp	Msat	Haplotype	Network Gp	Clade
EMR46	*T. alpinus*	Upper Lyell Canyon , YNP	37.76072	-119.25923	T.alp-N	Yes	Hap_1	North2	RED
217189	*T. alpinus*	Upper Lyell Canyon, YNP	37.76358	-119.26	T.alp-N	Yes	Hap_1	North2	RED
EMR72	*T. alpinus*	Gaylor Lakes, YNP	37.91122	-119.26625	T.alp-N	Yes	Hap_3*	North1	BLUE
EMR85	*T. alpinus*	Gaylor Lakes, YNP	37.91062	-119.26761	T.alp-N	Yes	Hap_3*	North1	BLUE
EMR91b	*T. alpinus*	Gaylor Lakes, YNP	37.91062	-119.26761	T.alp-N	Yes	Hap_3*	North1	BLUE
EMR82	*T. alpinus*	Gaylor Lakes, YNP	37.91008	-119.26835	T.alp-N	Yes	Hap_1	North2	RED
EMR76	*T. alpinus*	Gaylor Lakes, YNP	37.90986	-119.26875	T.alp-N	Yes	Hap_1	North2	RED
EMR81	*T. alpinus*	Gaylor Lakes, YNP	37.90912	-119.26948	T.alp-N	Yes	Hap_1	North2	RED
207203	*T. alpinus*	Evelyn Lake, YNP	37.80485	-119.3276	T.alp-N	Yes	Hap_6*	-	RED
207204	*T. alpinus*	Evelyn Lake, YNP	37.80485	-119.3276	T.alp-N	Yes	Hap_6*	-	RED
207205	*T. alpinus*	Evelyn Lake, YNP	37.80485	-119.3276	T.alp-N	Yes	Hap_6*	-	RED
207206	*T. alpinus*	Evelyn Lake, YNP	37.80485	-119.3276	T.alp-N	Yes	Hap_3*	North1	BLUE
207207	*T. alpinus*	Evelyn Lake, YNP	37.80485	-119.3276	T.alp-N	Yes	Hap_6*	-	RED
207199	*T. alpinus*	E end Fletcher Lake, YNP	37.79778	-119.3362	T.alp-N	Yes	Hap_3*	North1	BLUE
216271	*T. alpinus*	Upper Return Creek, Virginia Canyon, YNP	38.06129	-119.339	T.alp-N	Yes	Hap_3*	North1	BLUE
216272	*T. alpinus*	Upper Return Creek, Virginia Canyon, YNP	38.06129	-119.339	T.alp-N	Yes	Hap_7*	-	RED
222201	*T. alpinus*	Middle Young Lake, YNP	37.93876	-119.3405	T.alp-N	Yes	Hap_3*	North1	BLUE
222202	*T. alpinus*	Middle Young Lake, YNP	37.93876	-119.3405	T.alp-N	Yes	Hap_3*	North1	BLUE
222206	*T. alpinus*	Middle Young Lake, YNP	37.93876	-119.3405	T.alp-N	Yes	Hap_3*	North1	BLUE
JLP24337	*T. alpinus*	Middle Young Lake, YNP	37.93876	-119.3405	T.alp-N	Yes	Hap_3*	North1	BLUE
JLP24338	*T. alpinus*	Middle Young Lake, YNP	37.93876	-119.3405	T.alp-N	Yes	Hap_3*	North1	BLUE
JLP24345	*T. alpinus*	Middle Young Lake, YNP	37.93876	-119.3405	T.alp-N	Yes	Hap_3*	North1	BLUE
JLP24365	*T. alpinus*	Middle Young Lake, YNP	37.93876	-119.3405	T.alp-N	Yes	Hap_3*	North1	BLUE
JLP24373	*T. alpinus*	Middle Young Lake, YNP	37.93876	-119.3405	T.alp-N	Yes	Hap_9	North1	BLUE
JLP24374	*T. alpinus*	Middle Young Lake, YNP	37.93876	-119.3405	T.alp-N	Yes	Hap_3*	North1	BLUE
207209	*T. alpinus*	Vogelsang Lake, YNP	37.78769	-119.3469	T.alp-N	Yes	Hap_6*	-	RED
207210	*T. alpinus*	Fletcher Lake, YNP	37.78769	-119.3469	T.alp-N	Yes	Hap_6*	-	RED
207211	*T. alpinus*	Outlet Creek, Vogelsang Lake, YNP	37.78769	-119.3469	T.alp-N	Yes	Hap_6*	-	RED
219990	*T. alpinus*	Upper Cathedral Lake, YNP	37.84117	-119.4121	T.alp-N	Yes	Hap_3*	North1	BLUE
219991	*T. alpinus*	Upper Cathedral Lake, YNP	37.84117	-119.4121	T.alp-N	Yes	Hap_1	North2	RED
219992	*T. alpinus*	Upper Cathedral Lake, YNP	37.84117	-119.4121	T.alp-N	Yes	Hap_1	North2	RED
219993	*T. alpinus*	Upper Cathedral Lake, YNP	37.84117	-119.4121	T.alp-N	Yes	Hap_1	North2	RED
219994	*T. alpinus*	Upper Cathedral Lake, YNP	37.84117	-119.4121	T.alp-N	Yes	Hap_3*	North1	BLUE
219995	*T. alpinus*	Upper Cathedral Lake, YNP	37.84117	-119.4121	T.alp-N	Yes	Hap_1	North2	RED
219998	*T. alpinus*	Lower Ottaway Lake, YNP	37.64537	-119.4198	T.alp-N	Yes	Hap_1	North2	RED
220000	*T. alpinus*	Lower Ottaway Lake, YNP	37.64537	-119.4198	T.alp-N	Yes	Hap_1	North2	RED
220001	*T. alpinus*	Lower Ottaway Lake, YNP	37.64537	-119.4198	T.alp-N	Yes	Hap_1	North2	RED
220003	*T. alpinus*	Lower Ottaway Lake, YNP	37.64537	-119.4198	T.alp-N	Yes	Hap_1	North2	RED
220004	*T. alpinus*	Lower Ottaway Lake, YNP	37.64537	-119.4198	T.alp-N	Yes	Hap_1	North2	RED
220005	*T. alpinus*	Lower Ottaway Lake, YNP	37.64537	-119.4198	T.alp-N	Yes	Hap_1	North2	RED
220006	*T. alpinus*	Lower Ottaway Lake, YNP	37.64537	-119.4198	T.alp-N	Yes	Hap_1	North2	RED

Cat. No.	Species	Location	Lattitude	Longnitude	GeogGrp	Msat	Haplotype	Network Gp	Clade
216690	*T. alpinus*	Kerrick Meadow, YNP	38.11129	-119.4818	T.alp-N	Yes	Hap_3*	North1	BLUE
EMR69	*T. alpinus*	Mt. Hoffman; Northwest of May Lake, YNP	37.85449	-119.49239	T.alp-N	Yes	Hap_3*	North1	BLUE
EMR66	*T. alpinus*	Mt. Hoffman; Northwest of May Lake, YNP	37.85478	-119.49276	T.alp-N	Yes	Hap_3*	North1	BLUE
EMR71	*T. alpinus*	Mt. Hoffman; Northwest of May Lake, YNP	37.85548	-119.49289	T.alp-N	Yes	Hap_3*	North1	BLUE
23344	*T. alpinus*	Ten Lakes Trail above Ten Lakes, YNP	37.90135	-119.49807	T.alp-N	No	Hap_10	North2	RED
23345	*T. alpinus*	ridge near Ten Lakes, YNP	37.90135	-119.49807	T.alp-N	No	Hap_12	-	RED
EMR62	*T. alpinus*	Ten Lakes Pass, YNP	37.90510	-119.53470	T.alp-N	Yes	Hap_3*	North1	BLUE
EMR52	*T. alpinus*	Ten Lakes Pass, YNP	37.90290	-119.53505	T.alp-N	Yes	Hap_3*	North1	BLUE
EMR53	*T. alpinus*	Ten Lakes Pass, YNP	37.90290	-119.53505	T.alp-N	Yes	Hap_5	North1	BLUE
216306	*T. minimus*	Badger Flat	36.97099	-118.0954	T.min -Wh	Yes	Hap_27*	-	RED
216307	*T. minimus*	Badger Flat	36.97099	-118.0954	T.min -Wh	Yes	Hap_27*	-	RED
216308	*T. minimus*	Badger Flat	36.97099	-118.0954	T.min -Wh	Yes	Hap_27*	-	RED
216309	*T. minimus*	Badger Flat	36.97099	-118.0954	T.min -Wh	Yes	Hap_27*	-	RED
14976	*T. minimus*	Little Brush Meadow, Olancha Peak, Sierra Nevada	36.26623	-118.11771	T.min-S	Yes	Hap_41	South2	RED
224487	*T. minimus*	Little Brush Meadow, Olancha Peak, Inyo National Forest	36.25424	-118.1323	T.min-S	Yes	Hap_30	South2	RED
224485	*T. minimus*	Little Brush Meadow, Olancha Peak, Inyo National Forest	36.25278	-118.1336	T.min-S	Yes	Hap_30	South2	RED
224486	*T. minimus*	Little Brush Meadow, Olancha Peak, Inyo National Forest	36.25278	-118.1336	T.min-S	Yes	Hap_31	South2	RED
217106	*T. minimus*	N. Fork Crooked Creek	37.50348	-118.1476	T.min-C	Yes	Hap_27*	-	RED
217107	*T. minimus*	N. Fork Crooked Creek	37.50348	-118.1476	T.min-C	Yes	Hap_27*	-	RED
217111	*T. minimus*	N. Fork Crooked Creek	37.50348	-118.1476	T.min-C	Yes	Hap_27*	-	RED
217112	*T. minimus*	N. Fork Crooked Creek	37.50348	-118.1476	T.min-C	Yes	Hap_27*	-	RED
217113	*T. minimus*	N. Fork Crooked Creek	37.50348	-118.1476	T.min-C	Yes	Hap_27*	-	RED
217114	*T. minimus*	N. Fork Crooked Creek	37.50348	-118.1476	T.min-C	Yes	Hap_27*	-	RED
217116	*T. minimus*	N. Fork Crooked Creek	37.50348	-118.1476	T.min-C	Yes	Hap_27*	-	RED
217117	*T. minimus*	N. Fork Crooked Creek	37.50348	-118.1476	T.min-C	Yes	Hap_27*	-	RED
217118	*T. minimus*	N. Fork Crooked Creek	37.50348	-118.1476	T.min-C	Yes	Hap_44	-	RED
219907	*T. minimus*	south fork Cottonwood Creek, White Mts.	37.53101	-118.1651	T.min-C	Yes	Hap_27*	-	RED
219908	*T. minimus*	south fork Cottonwood Creek, White Mts.	37.53101	-118.1651	T.min-C	Yes	Hap_44	-	RED
219909	*T. minimus*	south fork Cottonwood Creek, White Mts.	37.53101	-118.1651	T.min-C	Yes	Hap_27*	-	RED
219910	*T. minimus*	south fork Cottonwood Creek, White Mts.	37.53101	-118.1651	T.min-C	Yes	Hap_27*	-	RED
219911	*T. minimus*	south fork Cottonwood Creek, White Mts.	37.53101	-118.1651	T.min-C	Yes	Hap_45	North1	BLUE
222659	*T. minimus*	Broder/Monache Meadow	36.159921	-118.1874	T.min-S	Yes	Hap_36	South2	RED
222660	*T. minimus*	Broder/Monache Meadow	36.159921	-118.1874	T.min-S	Yes	Hap_36	South2	RED
222661	*T. minimus*	Broder/Monache Meadow	36.162908	-118.1914	T.min-S	Yes	Hap_37	South2	RED
222662	*T. minimus*	Broder/Monache Meadow	36.162908	-118.1914	T.min-S	Yes	Hap_36	South2	RED
222663	*T. minimus*	Broder/Monache Meadow	36.162908	-118.1914	T.min-S	Yes	Hap_37	South2	RED
222664	*T. minimus*	Broder/Monache Meadow	36.162908	-118.1914	T.min-S	Yes	Hap_37	South2	RED
222665	*T. minimus*	Broder/Monache Meadow	36.162908	-118.1914	T.min-S	Yes	Hap_37	South2	RED
222666	*T. minimus*	Broder/Monache Meadow	36.175368	-118.2053	T.min-S	Yes	Hap_37	South2	RED
222667	*T. minimus*	Broder/Monache Meadow	36.175368	-118.2053	T.min-S	Yes	Hap_37	South2	RED
222668	*T. minimus*	Broder/Monache Meadow	36.175368	-118.2053	T.min-S	Yes	Hap_38	South2	RED

Cat. No.	Species	Location	Lattitude	Longnitude	GeogGrp	Msat	Haplotype	Network Gp	Clade
222669	*T. minimus*	Broder/Monache Meadow	36.175368	-118.2053	T.min-S	Yes	Hap_37	South2	RED
222670	*T. minimus*	Broder/Monache Meadow	36.175368	-118.2053	T.min-S	Yes	Hap_37	South2	RED
222671	*T. minimus*	Broder/Monache Meadow	36.175368	-118.2053	T.min-S	Yes	Hap_37	South2	RED
222672	*T. minimus*	Broder/Monache Meadow	36.175368	-118.2053	T.min-S	Yes	Hap_36	South2	RED
222673	*T. minimus*	Broder/Monache Meadow	36.175368	-118.2053	T.min-S	Yes	Hap_37	South2	RED
JAC399	*T. minimus*	Broder/Monache Meadow	36.175368	-118.2053	T.min-S	Yes	Hap_37	South2	RED
JAC401	*T. minimus*	Broder/Monache Meadow	36.175368	-118.2053	T.min-S	Yes	Hap_36	South2	RED
JAC403	*T. minimus*	Broder/Monache Meadow	36.175368	-118.2053	T.min-S	Yes	Hap_37	South2	RED
JAC404	*T. minimus*	Broder/Monache Meadow	36.175368	-118.2053	T.min-S	Yes	Hap_37	South2	RED
JAC405	*T. minimus*	Broder/Monache Meadow	36.175368	-118.2053	T.min-S	Yes	Hap_38	South2	RED
JAC412	*T. minimus*	Broder/Monache Meadow	36.175368	-118.2053	T.min-S	Yes	Hap_37	South2	RED
JAC413	*T. minimus*	Broder/Monache Meadow	36.175368	-118.2053	T.min-S	Yes	Hap_36	South2	RED
JAC415	*T. minimus*	Broder/Monache Meadow	36.175368	-118.2053	T.min-S	Yes	Hap_37	South2	RED
JAC417	*T. minimus*	Broder/Monache Meadow	36.175368	-118.2053	T.min-S	Yes	Hap_36	South2	RED
JAC418	*T. minimus*	Broder/Monache Meadow	36.175368	-118.2053	T.min-S	Yes	Hap_36	South2	RED
KCR487	*T. minimus*	Broder/Monache Meadow	36.175368	-118.2053	T.min-S	Yes	Hap_37	South2	RED
217271	*T. minimus*	Silver Canyon	37.40205	-118.2249	T.min-C	Yes	Hap_27*	-	RED
221244	*T. minimus*	below Big Pine Creek Campground, Sierra Nevada, Inyo Co	37.12927	-118.4059	T.min -Wh	Yes	Hap_47	-	RED
221274	*T. minimus*	Wier Lake, Sierra Nevada, Inyo Co.	37.17597	-118.563	T.min -Wh	Yes	Hap_27*	-	RED
221275	*T. minimus*	Wier Lake, Sierra Nevada, Inyo Co.	37.17597	-118.563	T.min -Wh	Yes	Hap_27*	-	RED
221260	*T. minimus*	South Lake, Sierra Nevada, Inyo Co.	37.17082	-118.5656	T.min -Wh	Yes	Hap_27*	-	RED
221263	*T. minimus*	South Lake, Sierra Nevada, Inyo Co.	37.17082	-118.5656	T.min -Wh	Yes	Hap_46	-	RED
221266	*T. minimus*	South Lake, Sierra Nevada, Inyo Co.	37.17082	-118.5656	T.min -Wh	Yes	Hap_47	-	RED
221270	*T. minimus*	South Lake, Sierra Nevada, Inyo Co.	37.17082	-118.5656	T.min -Wh	Yes	Hap_47	-	RED
221276	*T. minimus*	south end Adobe Valley, Mono Co.	37.792	-118.5675	T.min-N	Yes	Hap_48	-	RED
221277	*T. minimus*	south end Adobe Valley, Mono Co.	37.792	-118.5675	T.min-N	Yes	Hap_33	North1	BLUE
221278	*T. minimus*	south end Adobe Valley, Mono Co.	37.792	-118.5675	T.min-N	Yes	Hap_34	North1	BLUE
221247	*T. minimus*	South Fork Bishop Creek, Sierra Nevada, Inyo Co.	37.20996	-118.5691	T.min -Wh	Yes	Hap_27*	-	RED
221249	*T. minimus*	South Fork Bishop Creek, Sierra Nevada, Inyo Co.	37.20996	-118.5691	T.min -Wh	Yes	Hap_27*	-	RED
221251	*T. minimus*	South Fork Bishop Creek, Sierra Nevada, Inyo Co.	37.20996	-118.5691	T.min -Wh	Yes	Hap_27*	-	RED
221252	*T. minimus*	South Fork Bishop Creek, Sierra Nevada, Inyo Co.	37.20996	-118.5691	T.min -Wh	Yes	Hap_27*	-	RED
224150	*T. minimus*	Pine Creek, Sierra Nevada	37.36174	-118.6902	T.min -Wh	Yes	Hap_27*	-	RED
224151	*T. minimus*	Rock Creek, Sierra Nevada	37.44426	-118.7441	T.min-C	Yes	Hap_27*	-	RED
224152	*T. minimus*	Rock Creek, Sierra Nevada	37.44426	-118.7441	T.min-C	Yes	Hap_27*	-	RED
224153	*T. minimus*	Rock Creek, Sierra Nevada	37.44426	-118.7441	T.min-C	Yes	Hap_27*	-	RED
208328	*T. minimus*	Mono Mills	37.88811	-118.9602	T.min-N	Yes	Hap_40	-	RED
208329	*T. minimus*	Mono Mills	37.88811	-118.9602	T.min-N	Yes	Hap_40	-	RED
208330	*T. minimus*	Mono Mills	37.88811	-118.9602	T.min-N	Yes	Hap_33	North1	BLUE
208331	*T. minimus*	Mono Mills	37.88811	-118.9602	T.min-N	Yes	Hap_40	-	RED
208332	*T. minimus*	Mono Mills	37.88811	-118.9602	T.min-N	Yes	Hap_7*	-	RED
202384	*T. minimus*	William's Butte	37.90753	-119.1285	T.min-N	Yes	Hap_33	North1	BLUE

Cat. No.	Species	Location	Lattitude	Longnitude	GeogGrp	Msat	Haplotype	Network Gp	Clade
219225	*T. minimus*	William's Butte	37.90753	-119.1285	T.min-N	Yes	Hap_7*	-	RED
219226	*T. minimus*	William's Butte	37.90753	-119.1285	T.min-N	Yes	Hap_42	North1	BLUE
219229	*T. minimus*	William's Butte	37.90753	-119.1285	T.min-N	Yes	Hap_40	-	RED
219230	*T. minimus*	William's Butte	37.90753	-119.1285	T.min-N	Yes	Hap_40	-	RED
219231	*T. minimus*	William's Butte	37.90753	-119.1285	T.min-N	Yes	Hap_40	-	RED
219232	*T. minimus*	William's Butte	37.90753	-119.1285	T.min-N	Yes	Hap_43	North1	BLUE
219233	*T. minimus*	William's Butte	37.90753	-119.1285	T.min-N	Yes	Hap_40	-	RED
208551	*T. minimus*	Bohler Creek	37.90028	-119.1298	T.min-N	Yes	Hap_6*	-	RED
208554	*T. minimus*	Bohler Creek	37.90028	-119.1298	T.min-N	Yes	Hap_39	South2	RED
216299	*T. minimus*	Bohler Creek	37.90028	-119.1298	T.min-N	Yes	Hap_33	North1	BLUE
216302	*T. minimus*	Bohler Creek	37.90028	-119.1298	T.min-N	Yes	Hap_33	North1	BLUE
216303	*T. minimus*	Bohler Creek	37.90028	-119.1298	T.min-N	Yes	Hap_33	North1	BLUE
216304	*T. minimus*	Bohler Creek	37.90028	-119.1298	T.min-N	Yes	Hap_40	-	RED
221279	*T. minimus*	Sawmill Canyon, Sierra Nevada, Mono Co.	37.87559	-119.1416	T.min-N	Yes	Hap_7*	-	RED
221280	*T. minimus*	Sawmill Canyon, Sierra Nevada, Mono Co.	37.87559	-119.1416	T.min-N	Yes	Hap_40	-	RED
224154	*T. minimus*	Swager Canyon, Sweetwater Mts.	38.38664	-119.3446	T.min-N	Yes	Hap_3*	North1	BLUE
224140	*T. minimus*	Sonora Pass	38.32799	-119.6372	T.min-N	Yes	Hap_32	North1	BLUE
224141	*T. minimus*	Sonora Pass	38.32799	-119.6372	T.min-N	Yes	Hap_3*	North1	BLUE
224142	*T. minimus*	Sonora Pass	38.32799	-119.6372	T.min-N	Yes	Hap_33	North1	BLUE
224143	*T. minimus*	Sonora Pass	38.32799	-119.6372	T.min-N	Yes	Hap_32	North1	BLUE
224144	*T. minimus*	Sonora Pass	38.32799	-119.6372	T.min-N	Yes	Hap_34	North1	BLUE
224145	*T. minimus*	Sonora Pass	38.32799	-119.6372	T.min-N	Yes	Hap_32	North1	BLUE
224146	*T. minimus*	Sonora Pass	38.32799	-119.6372	T.min-N	Yes	Hap_32	North1	BLUE
224147	*T. minimus*	Sonora Pass	38.32799	-119.6372	T.min-N	Yes	Hap_32	North1	BLUE
224148	*T. minimus*	Sonora Pass	38.32799	-119.6372	T.min-N	Yes	Hap_35	North2	RED
224149	*T. minimus*	Sonora Pass	38.32799	-119.6372	T.min-N	Yes	Hap_32	North1	BLUE

* haplotypes shared by both species

\- no labeled network group; see Figure 4 in main text

N/A: haplotype more than 12 steps from network
