# Supplementary material for: Diversification of the Alpine Chipmunk, Tamias alpinus, an alpine endemic of the Sierra Nevada, California
Source: BMC Evol Biol. 2014 Feb 23;14:34. doi: 10.1186/1471-2148-14-34 (PMC4077034; doi:10.1186/1471-2148-14-34)
Supplement: Additional file 3: Table S2 — Microsatellite and cytochrome b primers used in analyses. [file 1471-2148-14-34-S3.pdf]

Supplementary Information

Recent speciation or reticulate evolution? Diversification of the Alpine Chipmunk, *Tamias alpinus,* an alpine endemic of the Sierra Nevada, California.

Emily M. Rubidge, James L. Patton and Craig Moritz

Table S2. Microsatellite primers used in analyses. *Tamias* specific cytochrome b primer sequences developed for museum skin specimens collected between 1911-1916; fragments were pieced together to make up 780bp product.

Primer	Sequence or Reference	Repeat Motif	T_a	Range
Microsatellite Primers				
Eu26	Schulte-Hostedde et al 2000	$(CA)_{20}$	55	140-200
Eu37	Schulte-Hostedde et al 2000	$(GA)_{17}$	55	120-160
Eu41	Schulte-Hostedde et al 2000	$(GT)_{16}$	55	120-160
Eu86	Schulte-Hostedde et al 2000	$(AC)_{21}$	55	140-180
Eu94	Schulte-Hostedde et al 2000	$(GT)_{14}$	51	90-140
AC A2	F 5'-ACA TCC ACT TTA CTC CCC A AC TC-3'	$(TTTC)_9(TAC)_8$	60	240-340
	R 5' AGA GGC TAT GTC AGG AGG ATC'3			
AC B111	F 5'- AAT ACC TAT CCA CCC ATC ATC -3'	$(GTCT)_8(CT)_6(CA)_{16}$	57	230-245
	R 5' TTC CCT CAA GCA GTC ATA AG -3'			
AC B12	F 5'-AAA GGA ACC CTC TAC ACC AC -3'	$(TG)_{11}(TCTG)_7$	58	250-300
	R 5'-ACC GAA ACC ACT CAG AAC AG -3'			
AC A101	F 5' CTA GCC TCA GCC ACT CAG TG -3'	$(TAG)_{10}(AAT)_6$	58	120-190
	R 5'- GGT TCT ACC TTT GAG CCA CAC -3'			
AC A108	F 5'- CTG CTG CCT CTT CTT CTT C-3'	$(TAC)_{18}$	60	250-280
	R 5' CCC ATA ATC CCA GTG ACT C- 3'			
AC C2	F 5' CTG GGA ACA TAG TCT TTA GGC -3'	$(ATCC)_8$	60	110-160
	R 5' GTG AAT GGG CAG ATG ATT AG -3'			
AC D115	F 5' CTT TGC CAC ATC TGA CTT G -3'	$(TCTA)_9$	58	160-200
	R 5' ACT GGG TAG ACT GAG AAA TAC G -3'			
AC D107	F 5' TAA TTC AAC TCT GGG AAC ACT G -3'	$(TATC)_{10}$	57	170-200
	R 5' GAA AGG AGC CTA AGC TGT TG -3'			
AC C122	F 5' CCC ATC CAT CTA TCT GTT CAT C-3'	$(TA)_3TT(TCCA)_9$	58	100-190
Tamias specific cytochrome b primers		**Product size (bp)**		
TAMH4F	ATG ACA AAC ATC CGC AAA AC			
TAMH3R	TCC GTT AGC GTG TAT GTA TCG	239	50	
TAMH5F	CCG ATA CAT ACA CGC CAA CG			
TAMH2R	TCC TCA TGG GAG AAC GTA GC	171	50	
TAMH6F	GCT ACG TTC TCC CAT GAG GA			
TAMH6R	TCC TTG GAG ACC CAG ACA AC	390	50	
