# Supplementary material for: Diversification of the Alpine Chipmunk, Tamias alpinus, an alpine endemic of the Sierra Nevada, California
Source: BMC Evol Biol. 2014 Feb 23;14:34. doi: 10.1186/1471-2148-14-34 (PMC4077034; doi:10.1186/1471-2148-14-34)
Supplement: Additional file 4 — Detailed results and additional figures for morphological analyses. [file 1471-2148-14-34-S4.pdf]

Supplementary Information

Recent speciation or reticulate evolution? Diversification of the Alpine Chipmunk, *Tamias alpinus,* an alpine endemic of the Sierra Nevada, California.

Emily M. Rubidge, James L. Patton and Craig Moritz

Results of Morphological Analyses:

Table S3. Sample Sizes for Morphological Analyses

Species	External Body Metrics	Craniodental characteristics	Balcular Measurements
T. alpinus	36	161	15
T. minimus	129	159	33

1. Bacular analyses

For the analysis of bacular morphology, our sample size was limited as follows: n=15 for *T. alpinus* (12 A-north, 3 B-south) and n=33 for *T. m. scrutator* (18 C-Sierras, 16 E-White Mts). Five variables were measured including shaft length, mid-shaft height, tip height, tip angle, and keep breadth; see Patterson and Thaeler 1982 for measurement protocol). The two species differ significantly ($p < 0.05$, one-way ANOVA) in all characters except tip angle, with *alpinus* having a notably shorter baculum (mean shaft length, respectively by sample, 2.87-2.94 mm versus 3.64-3.66 mm) but longer tip (0.647-0.687 mm versus 0.590-0.608 mm). The CVA correctly classified all specimens to their respective species membership, even through there were misclassifications within each species relative to their geographic samples. The posterior probabilities of group membership for all specimens, with a single exception, were > 0.9928. The single exception was a specimen of *T. minimus* from tgroup C-Sierras with a posterior probability of 0.7070; this specimen thus has a baculum more similar to that of *alpinus* than other *minimus* (Figure 2 in main manuscript and Figure S1)

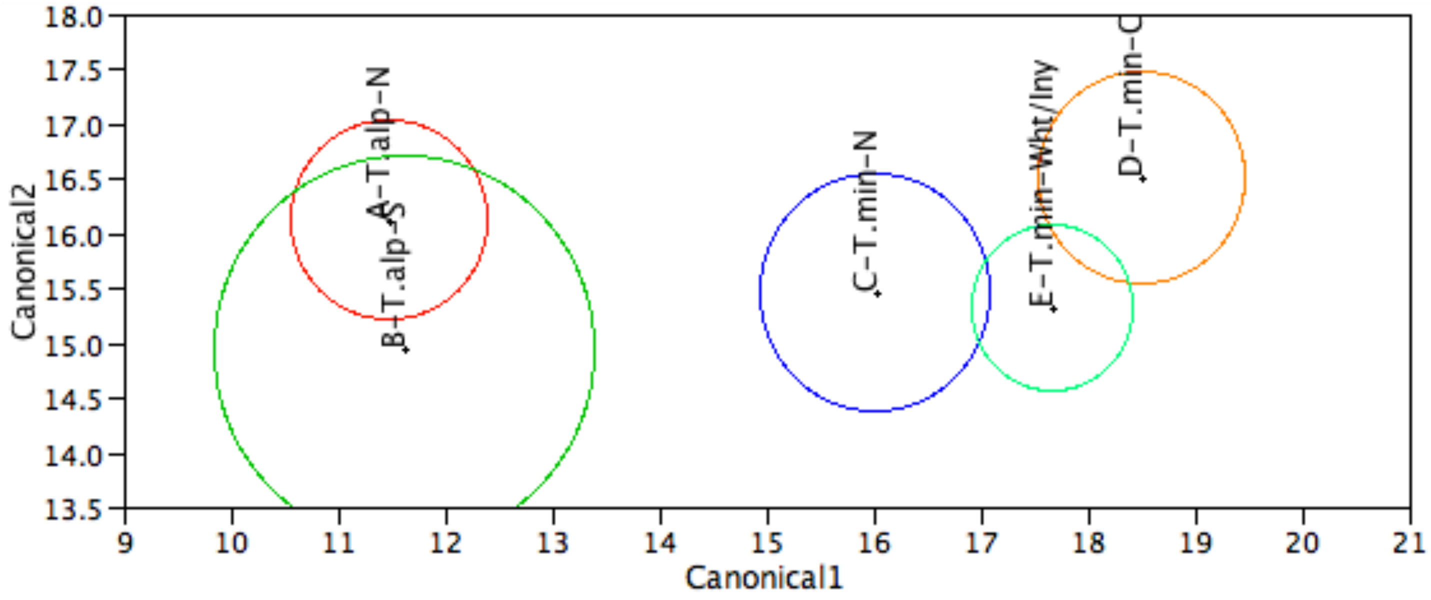

Figure S1. Bivariate plot of the first and second canonical variates scores based on 5 bacular measurements; 95% confidence limits are plotted for two geographic samples each of *Tamias alpinus* (A, T.alp-N; B, T.alp-S; see main text Fig. 1) and *T. minimus* (C, T.min-N; D-T.min-C; E, T.min-Wht/Iny). Shaft length contributes most strongly to separation of CV-1, which explains 94.1% of the total pool of variation); tip height to CV-2 (3.1% of the total variation).

2. Body Metrics

The results of the analyses of external characters (total length, tail length, ear height, tail hair length ['bushiness']) showed that *T. minimus* has significantly longer and narrower tails than *T. alpinus* and shorter ears ($p < 0.0001$) in all comparisons Table S2.

Table S4. Measurements of external characters in *T. minimus* and *T. alpinus* in study area

Species	Tail length (mm)	Tail hair length (mm)	Ear length (mm)
T. minimus	79.9-82.1	12.9-14,44	14.6-15.2
T. alpinus	74.9-76.7	16.0-16,5	16.6-17.0

The two species are strongly separable in the CVA analysis of external characters, with tail "bushiness" contributing to most of the variation along CV-1, which accounts for nearly all of the variation (99.09%; Figure S2). Misclassification of individuals, however, is present, as 3 of 36 *alpinus* (8.3%) were misclassified as *minimus,* equally distributed to each of the three geographic samples of *minimus* with posterior probabilities of group membership of 0.4932 or greater. Alternatively, 5 of 129 (3.9%) *minimus* were misclassified as *alpinus*. Two *minimus* from the T.min-N sample were misclassified as *alpinus* from the adjacent T.alp-N sample, or combined T.alp-N and T.alp-S samples, at posterior probabilities of 0.5064 and 0.8425, respectively; one T.min-C individual was group with the T.alp-N sample at a posterior probabilities of 0.4984; and two individuals from the T.min-Wht/Iny sample were misclassified as T.alp-S, at posterior probabilities of 0.4836 and 0.6269. Misclassified specimens in both directions were rather intermediate in their external characters relative to each a priori group.

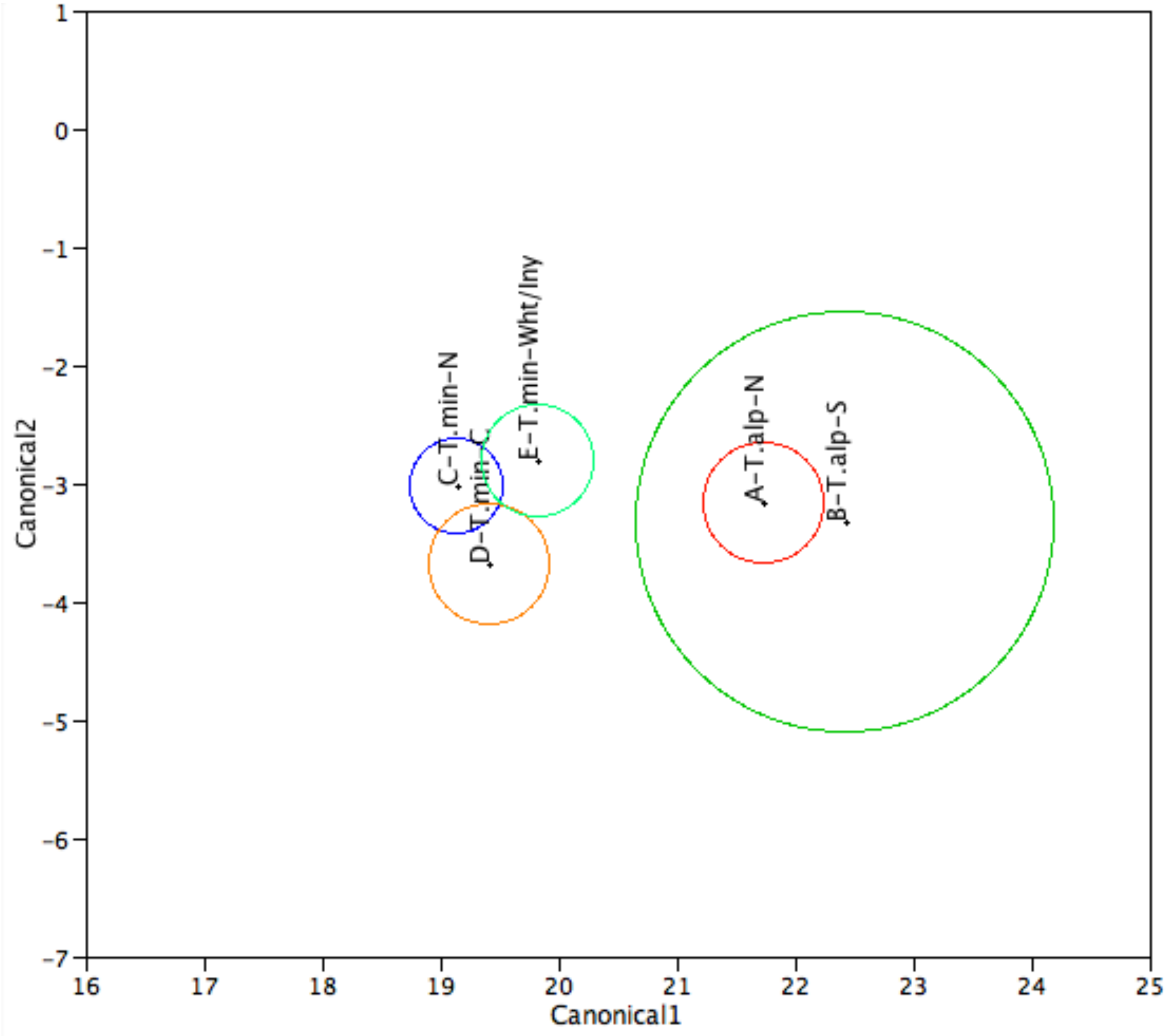

Figure S2. Bivariate plot of the first and second canonical variates scores based on 3 external measurements; 95% confidence limits are plotted for two geographic samples each of *Tamias alpinus* (A, T.alp-N; B, T.alp-S; see main text Fig. 1) and *T. minimus scrutator* (C, T.min-N; D-T.min-C; E, T.min-Wht/Iny). CV-1 explains 93.5% of the total pool of variation; CV-2 explains 6.19%.

3. Craniodental characters

The analyses of craniodental characters included 21 linear measurements of standard skull and mandibular length, width, and height variables. The two species and their respective geographic samples, are sharply differentiated in multivariate space by measured craniodental and mandibular variables (Figure S3)

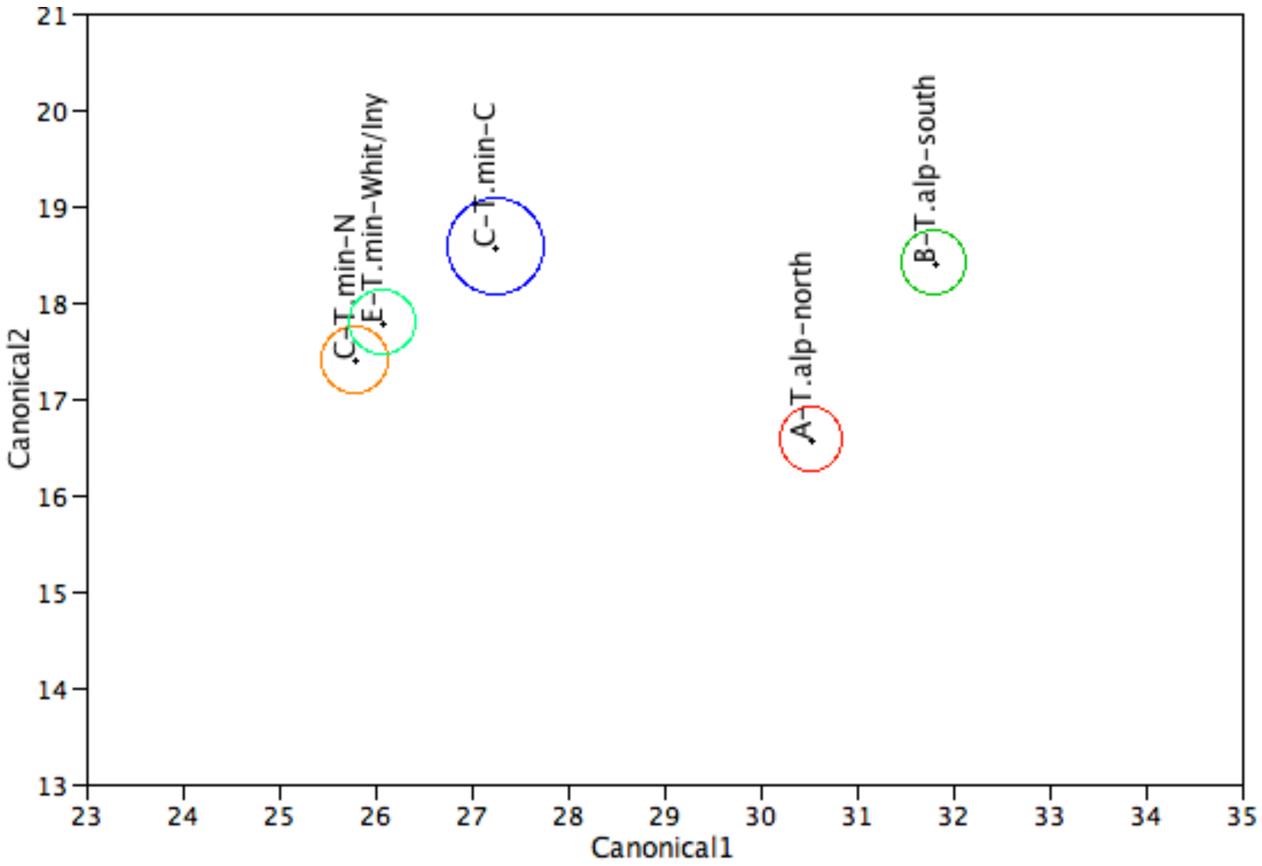

Figure S3. Bivariate plot of the first and second canonical variates scores based on craniodental measurements; 95% confidence limits are plotted for two geographic samples each of *Tamias alpinus* (A, T.alp-N; B, T.alp-S; see main text Fig. 1) and *T. minimus* (C, T.min-N; D-T.min-C; E, T.min-Wht/Iny). CV-1 explains 85.61% of the total pool of variation; CV-2 explains 7.22%.

Two of 161 (1.2%) *alpinus* are misclassified as *minimus* (one specimen each from the T.alp-N sample, both placed in the T.min-C sample at posterior probabilities of 0.5126 and 0.7117; and 2 of 159 (1.1%) *minimus* from the T.min-C sample are misclassified as *alpinus*, one to T.alp-N, with posterior probability of 0.5090, and the other to T.alp-S, posterior probability of 0.5246. For comparison, misclassification rate is 15.1% (24 of 159) of individuals belonging to the two geographic samples of *alpinus* and 29.5% of *minimus* individuals are misclassified relative to the three samples of this taxon.
