# Supplementary material for: Diversification of the Alpine Chipmunk, Tamias alpinus, an alpine endemic of the Sierra Nevada, California
Source: BMC Evol Biol. 2014 Feb 23;14:34. doi: 10.1186/1471-2148-14-34 (PMC4077034; doi:10.1186/1471-2148-14-34)
Supplement: Additional file 5: Figure S4 — Bayesian estimate (GTR + I + Γ) of phylogeny of cyt b. [file 1471-2148-14-34-S5.pdf]

Results of gene tree analysis:

One clade comprised all geographic areas and both species (Red on Figure S4), whereas the other (Blue on Figure S4) comprised only individuals of both species from the northern sampling area and two individuals from the White Mountains. Both clades included haplotypes that were shared between species. There were two divergent haplotypes comprised of *T. alpinus* individuals from the southernmost portion of their range.

Figure S4. Bayesian estimate (GTR + I + Γ) of phylogeny of cyt b for 47 haplotypes of *T. alpinus*, *T. minimus* and *T. panamintinus* (outgroup). Posterior probabilities of major mtDNA clades are shown. The species that haplotypes belong, including shared haplotypes (AlpMin1, 2, 3, & 4), are labeled at tips. Map shows geographic location of each clade. The red solid circles represent individuals with haplotypes within the "red" clade. Blue open circles represent individuals with haplotypes within the "blue" clade and green triangles show the individuals with the divergent Alp-S haplotype (n=4) and the black triangle show the unique haplotype that is divergent from the other haplotypes. Scale represents expected changes per site. There is one *T. alpinus* individual from Bullfrog Lake (MVZ224480) in the T.alp-S geographic group that was assigned to T.Min-N based on its genotype at 14 loci

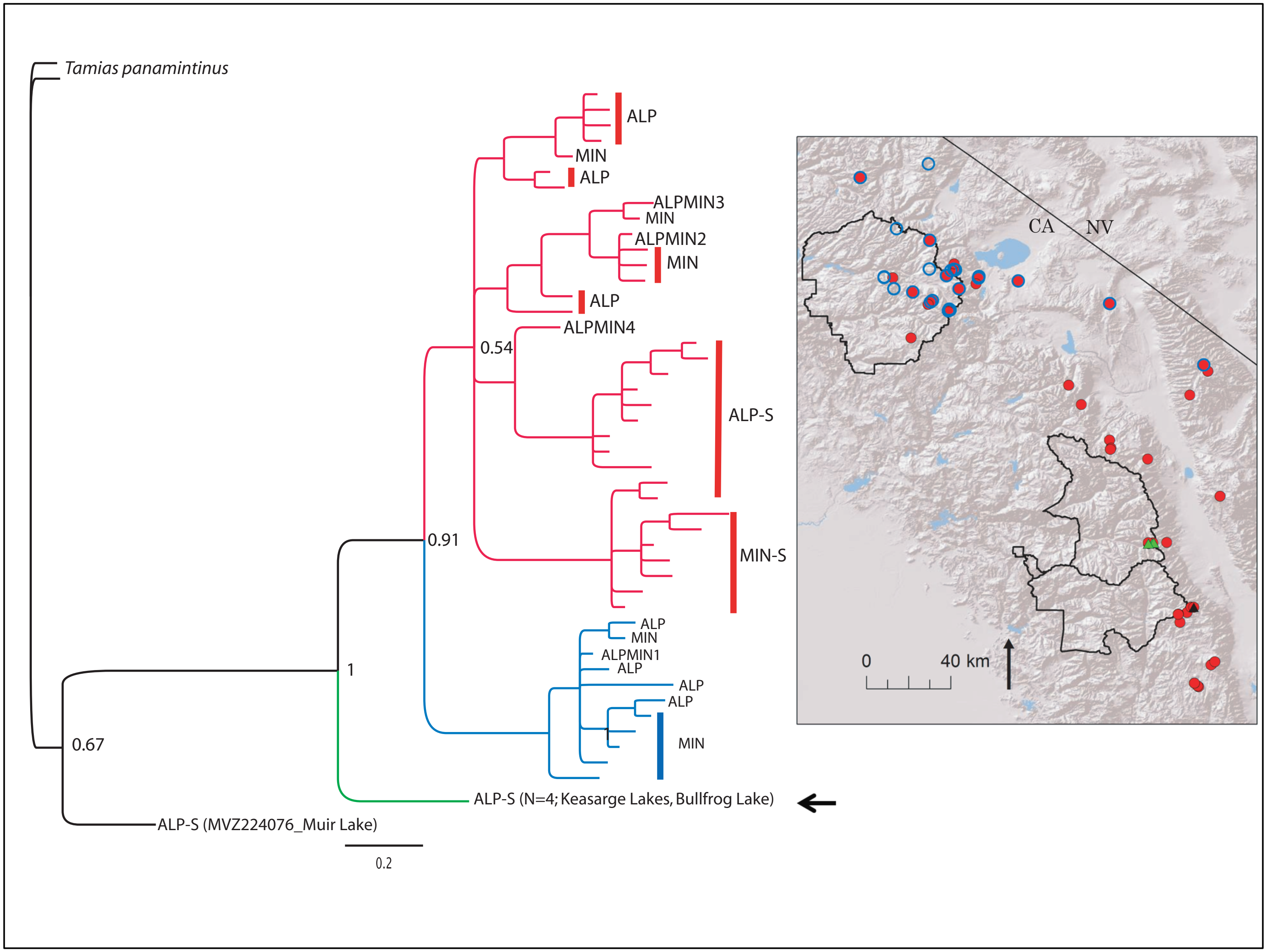
Tamias panamintinus
ALP
MIN
ALP
ALPMIN3
MIN
ALPMIN2
MIN
ALP
ALPMIN4
0.54
ALP-S
MIN-S
0.91
ALP
MIN
ALPMIN1
ALP
ALP
ALP
MIN
1
0.67
ALP-S (N=4; Keasarge Lakes, Bullfrog Lake)
ALP-S (MVZ224076_Muir Lake)
0.2
CA
NV
0
40 km
