# Supplementary material for: Diversification of the Alpine Chipmunk, Tamias alpinus, an alpine endemic of the Sierra Nevada, California
Source: BMC Evol Biol. 2014 Feb 23;14:34. doi: 10.1186/1471-2148-14-34 (PMC4077034; doi:10.1186/1471-2148-14-34)
Supplement: Additional file 6: Figure S5 — NJ tree of the relationships among geographic groups of T. alpinus and T. minimus based on the average number of nucleotide substitutions per site (Dxy, Nei [51]) at cyt b. [file 1471-2148-14-34-S6.pdf]

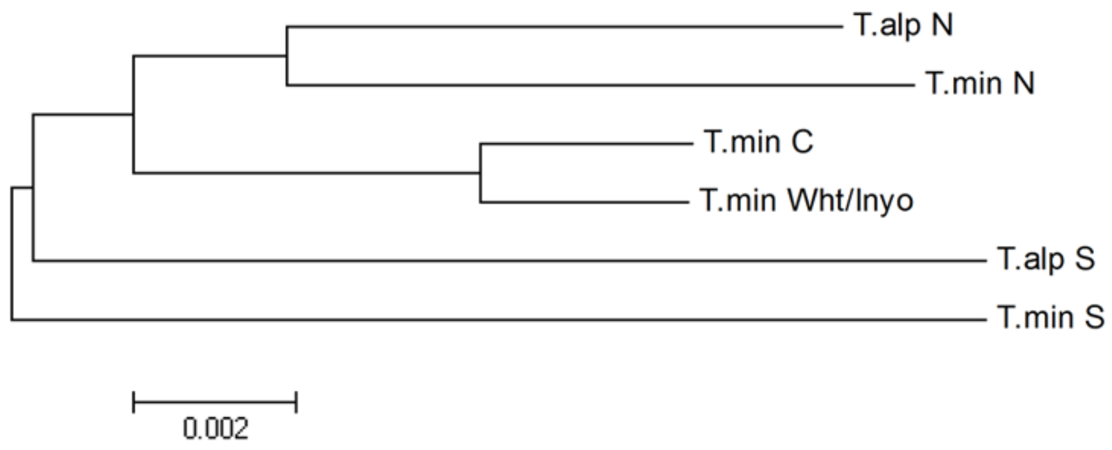

Figure S5. NJ tree of the relationships among geographic groups of *T. alpinus* and *T. minimus* based on the average number of nucleotide substitutions per site (Dxy, Nei 1987) at *cyt b*.
