# Supplementary material for: Diversification of the Alpine Chipmunk, Tamias alpinus, an alpine endemic of the Sierra Nevada, California
Source: BMC Evol Biol. 2014 Feb 23;14:34. doi: 10.1186/1471-2148-14-34 (PMC4077034; doi:10.1186/1471-2148-14-34)
Supplement: Additional file 8: Figure S6 — Estimation of the true number of clusters using ∆K (Evanno et al [62]) and Figure S7. Unrooted NJ tree of the relationship among populations using FST. [file 1471-2148-14-34-S8.pdf]

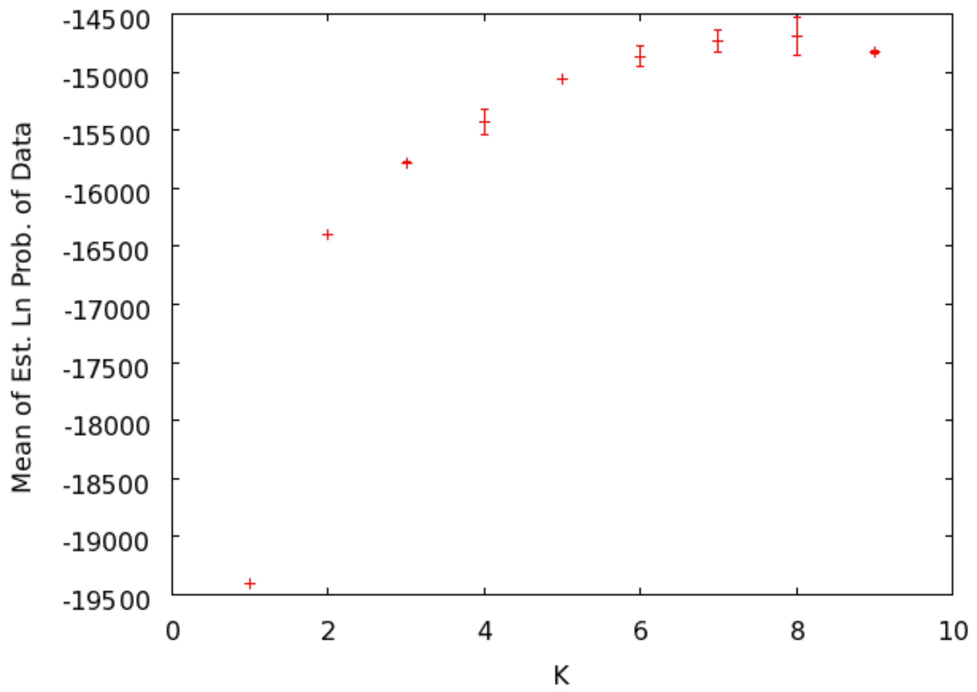

a)

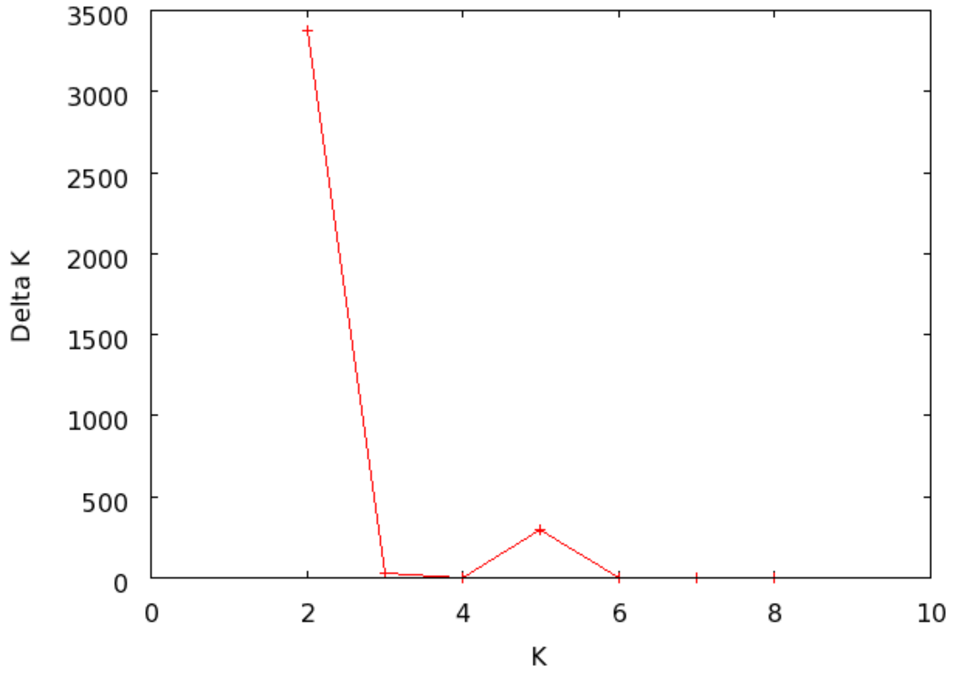

b)

Figure S6. Estimation of the true number of clusters using ΔK (Evanno et al 2005); a) Mean likelihood values (±SD) over 5 runs for each K, asymptotes at K=6; b) ΔK values, where uppermost level is the true number of clusters, which in this case is K=2.

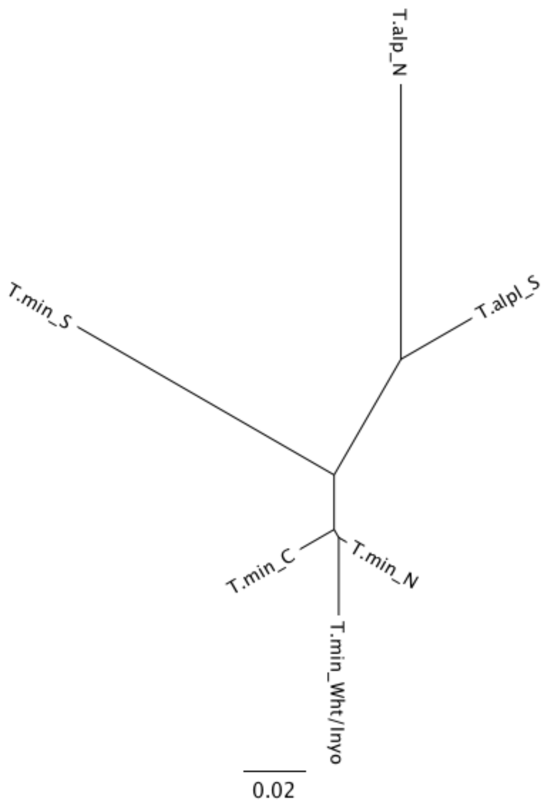

Figure S7. Unrooted NJ tree of the relationship among populations using F_{ST} (Weir and Cockerham 1984) based on 14 microsatellite loci.
